# Intermixing‐Driven Surface and Bulk Ferromagnetism in the Quantum Anomalous Hall Candidate MnBi_6_Te_10_

**DOI:** 10.1002/advs.202203239

**Published:** 2023-02-17

**Authors:** Abdul‐Vakhab Tcakaev, Bastian Rubrecht, Jorge I. Facio, Volodymyr B. Zabolotnyy, Laura T. Corredor, Laura C. Folkers, Ekaterina Kochetkova, Thiago R. F. Peixoto, Philipp Kagerer, Simon Heinze, Hendrik Bentmann, Robert J. Green, Pierluigi Gargiani, Manuel Valvidares, Eugen Weschke, Maurits W. Haverkort, Friedrich Reinert, Jeroen van den Brink, Bernd Büchner, Anja U. B. Wolter, Anna Isaeva, Vladimir Hinkov

**Affiliations:** ^1^ Physikalisches Institut (EP‐IV) Universität Würzburg Am Hubland D‐97074 Würzburg Germany; ^2^ Würzburg‐Dresden Cluster of Excellence ct.qmat Germany; ^3^ Leibniz Institut für Festkörper‐ und Werkstoffforschung (IFW) Dresden Helmholtzstraße 20 D‐01069 Dresden Germany; ^4^ Centro Atómico Bariloche Instituto de Nanociencia y Nanotecnología (CNEA‐CONICET) and Instituto Balseiro. Av. Bustillo 9500 Bariloche 8400 Argentina; ^5^ Institut für Festkörper‐ und Materialphysik Technische Universität Dresden D‐01062 Dresden Germany; ^6^ Physikalisches Institut (EP‐VII) Universität Würzburg Am Hubland D‐97074 Würzburg Germany; ^7^ Institute for Theoretical Physics Heidelberg University Philosophenweg 19 69120 Heidelberg Germany; ^8^ Department of Physics and Astronomy and Stewart Blusson Quantum Matter Institute University of British Columbia Vancouver British Columbia V6T 1Z4 Canada; ^9^ Department of Physics and Engineering Physics University of Saskatchewan Saskatoon SK S7N 5E2 Canada; ^10^ ALBA Synchrotron Light Source E‐08290 Cerdanyola del Vallès Barcelona Spain; ^11^ Helmholtz‐Zentrum Berlin für Materialien und Energie Albert‐Einstein‐Straße 15 D‐12489 Berlin Germany; ^12^ Institut für Theoretische Physik Technische Universität Dresden D‐01062 Dresden Germany; ^13^ Van der Waals‐Zeeman Institute Department of Physics and Astronomy University of Amsterdam Science Park 904 Amsterdam 1098 XH The Netherlands

**Keywords:** intrinsic magnetic topological insulator, magnetic topological insulator, magnetism, superconducting quantum interference device (SQUID), topological insulator, X‐ray absorption spectroscopy (XAS), X‐ray magnetic circular dichroism (XMCD)

## Abstract

The recent realizations of the quantum anomalous Hall effect (QAHE) in MnBi_2_Te_4_ and MnBi_4_Te_7_ benchmark the (MnBi_2_Te_4_)(Bi_2_Te_3_)_*n*_ family as a promising hotbed for further QAHE improvements. The family owes its potential to its ferromagnetically (FM) ordered MnBi_2_Te_4_ septuple layers (SLs). However, the QAHE realization is complicated in MnBi_2_Te_4_ and MnBi_4_Te_7_ due to the substantial antiferromagnetic (AFM) coupling between the SLs. An FM state, advantageous for the QAHE, can be stabilized by interlacing the SLs with an increasing number *n* of Bi_2_Te_3_ quintuple layers (QLs). However, the mechanisms driving the FM state and the number of necessary QLs are not understood, and the surface magnetism remains obscure. Here, robust FM properties in MnBi_6_Te_10_ (*n* = 2) with *T*_c_ ≈ 12 K are demonstrated and their origin is established in the Mn/Bi intermixing phenomenon by a combined experimental and theoretical study. The measurements reveal a magnetically intact surface with a large magnetic moment, and with FM properties similar to the bulk. This investigation thus consolidates the MnBi_6_Te_10_ system as perspective for the QAHE at elevated temperatures.

## Graphical abstract

Stabilizing the quantum anomalous Hall effect in a magnetic topological insulator such as MnBi_6_Te_10_ is a promising route toward topological qubits, ultra‐low‐power electronics and spintronics. The most fundamental ingredient is ferromagnetic order; however, in pristine MnBi_6_Te_10_, the consecutive Mn layers interact antiferromagnetically. Here, it is demonstrated how Mn and Bi intermixing between their respective atomic positions may induce robust ferromagnetism.

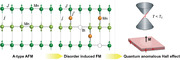

## 1 Introduction

Theory provides a seemingly straightforward avenue toward novel quantum effects such as the quantum anomalous Hall (QAH) effect,^[^ ^1^, ^2^, ^3^, ^4^, ^5^ ^]^ and axion electrodynamics,^[^ ^6^, ^7^, ^8^, ^9^ ^]^ namely to induce a long‐range ferromagnetic (FM) order in topological insulators (TI).^[^ ^10^, ^11^ ^]^ The vision of observing Majorana fermions and implementing topological qubits at superconductor/QAH insulator interfaces,^[^ ^12^ ^]^ ultra low‐power electronics,^[^ ^13^ ^]^ and applications in spintronics^[^ ^14^ ^]^ has ignited substantial experimental efforts in this direction. Yet, hitherto the QAH effect (QAHE) has only been demonstrated in the (sub‐)Kelvin range.^[^ ^3^, ^4^, ^15^ ^]^ The experimental realization of the QAHE is complicated by several simultaneous requirements to a candidate system: The Dirac point (DP) of the parent TI should be well within its bulk band gap; the chemical potential has to be tuned to the DP; the introduced magnetic subsystem should lead to a substantial surface ferromagnetism to open a large exchange gap at the DP; and the material's bulk should remain insulating.

**Figure 1:**
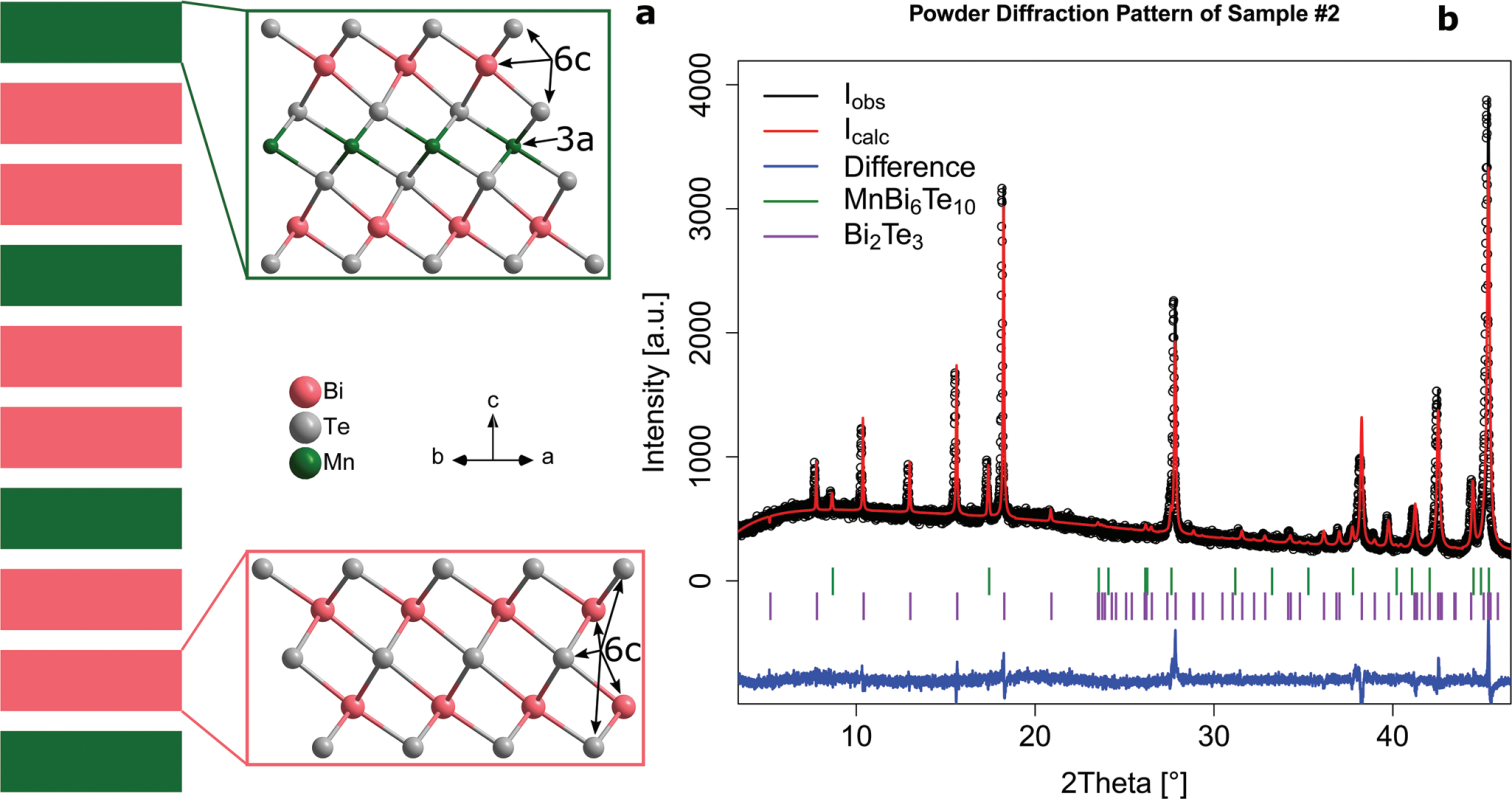
Crystal structure properties. a) The unit cell of MnBi_6_Te_10_ is sketched by slabs of red and green boxes, where green indicates a septuple layer and red indicates a quintuple layer. In the expanded views we show the atomic structure. The QLs and SLs are interleaved by van der Waals gaps. b) Experimental (black) and refined by Rietveld method (red) powder X‐ray diffraction pattern of the sample #2 in the 2θ range 5° − 45°. For the full 2θ range, see Figure S1, Supporting Information. The difference curve is shown in blue (*R*_p_ = 0.055, *wR*_p_ = 0.071, GoF = 1.48). A small fraction of Bi_2_Te_3_ comprises 7 wt% ($R\bar{3}m$, *a* = 4.3797(4) Å, *c* = 30.4965(7) Å, *R*_obs_ = 0.094, *wR*_obs_ = 0.096, *R*_all_ = 0.109). The main phase is refined with the overall Mn_0.8_Bi_6.2_Te_10_ composition ($R\bar{3}m$, *a* = 4.3667(2) Å, *c* = 101.869(4) Å, *R*_obs_ = 0.079, *wR*_obs_ = 0.074, *R*_all_ = 0.104).

The first materials to exhibit the QAHE were extrinsically doped (V/Cr)_*x*_(Bi,Sb)_2 − *x*_Te_3_, which consist of van‐der‐Waals coupled quintuple layers (QLs, see **Figure** **1**). However, band engineering by tuning the Bi/Sb ratio does not move the DP sufficiently above the valence band,^[^ ^16^ ^]^ V/Cr impurity bands overlap with the alleged exchange gap^[^ ^17^ ^]^ and residual bulk conductance destroys quantization with increasing temperature.^[^ ^18^ ^]^ As a result, the QAHE is stable only below *T*_QAH_ = 20 mK.

The intrinsic magnetic topological insulators (MnBi_2_Te_4_)(Bi_2_Te_3_)_*n*_ (MBT_*n*_, *n* = 0–4), whose functional constituents are (MnBi_2_Te_4_) septuple layers (SLs) with the central sheet of FM‐ordered Mn atoms, separated by *n* Bi_2_Te_3_ QLs, offer several advantages. Whereas for QL termination, angle‐resolved photoemission spectroscopy (ARPES) measurements on MBT_1_ and MBT_2_ yield similar results to (Bi,Sb)_2_Te_3_, without a discernible DP, there is a DP within the bulk gap for the SL termination.^[^ ^19^ ^]^ Also, the topmost sheet of the ferromagnetically arranged Mn moments should strongly couple with the topological surface states (TSS), albeit a thorough spectroscopic investigation of the surface magnetism is still pending. As a result, a much higher *T*_QAH_ = 1.4 K is achieved in MnBi_2_Te_4_.^[^ ^20^ ^]^ This is despite the fact that MnBi_2_Te_4_ is suboptimal due to its antiferromagnetic (AFM) order (*T*_N_ = 24 K) and a complex layer‐number dependence of the quantization effects, with an odd number of SLs required to realize the QAHE.^[^ ^20^ ^]^

Yet, the potential of the other MBT_*n*_ for a further substantial increase of *T*_QAH_ is strong. Increasing *n* weakens the interlayer AFM coupling so that FM properties gradually develop. Indeed, most studies report a complex metamagnetic behavior in MnBi_4_Te_7_ and MnBi_6_Te_10_,^[^ ^21^, ^22^, ^23^, ^24^, ^25^, ^26^, ^27^ ^]^ but a clear FM state only for *n* ⩾ 3.^[^ ^22^, ^23^, ^28^ ^]^ Already metamagnetic MnBi_4_Te_7_ hosts the QAHE up to several degree Kelvin in the bulk regime.^[^ ^29^ ^]^ This experimental realization of a QAHE device out of a bulk crystal required technically challenging but feasible efforts to attain the charge neutrality condition.^[^ ^29^ ^]^ Consequently, envisioning this clear, technical realization path once an appropriate bulk crystal exists, we focus here on the next obvious step, namely to strengthen the FM properties of MBT_*n*_. Our synthesis endeavors culminate in the robust FM order in MnBi_6_Te_10_ (i.e., already for *n* = 2), an intrinsic magnetic TI^[^ ^19^ ^]^ and QAHE candidate.^[^ ^30^ ^]^

We confirm the FM state both in the bulk and on the surface of MnBi_6_Te_10_ crystals by using bulk‐sensitive superconducting quantum interference device (SQUID) magnetometry and surface‐sensitive X‐ray magnetic circular dichroism (XMCD). The clear FM characteristics seemingly contradict the weak AFM coupling anticipated by our density functional theory (DFT) calculations for the atomically ordered compound. This disagreement is resolved by including the experimentally determined Mn substoichiometry and Mn/Bi site intermixing into account. Our calculations pinpoint that the magnetic coupling can be tuned toward ferromagnetism by appropriate intermixing already in MnBi_4_Te_7_ and even MnBi_2_Te_4_. Considering the intermixing patterns in our MnBi_6_Te_10_ samples and those reported showing no ferromagnetism, we rationalize their differing magnetic behavior. Our results demonstrate that carefully engineered intermixing can accomplish a robust FM order and, therefore, is the key toward enhanced QAHE properties in the MBT_*n*_ family of intrinsic magnetic topological insulators.

## 2 Results

### 2.1 Crystal Growth and Structure Refinement

MnBi_6_Te_10_ crystals were grown by slow crystallization from a melt (see Section 4). Besides MnBi_6_Te_10_, the obtained ingot contained admixtures of Bi_2_Te_3_ and MnTe_2_ (see Figure S1, Supporting Information). Observing side phases fully agrees with our earlier studies of MnBi_6_Te_10_ melting and decomposition by differential scanning calorimetry.^[^ ^31^ ^]^ Their occurrence can be related to crystal growth being a competitive process between MnBi_6_Te_10_, MnBi_8_Te_13_, and Bi_2_Te_3_, all having nearly the same crystallization temperatures.

A series of energy‐dispersive X‐ray spectroscopy (EDX) point measurements on individual crystals extracted from the ingot demonstrated a compositional range between Mn: 5.0, Bi: 36.6, Te: 58.4, and Mn: 4.2, Bi: 37.1, Te: 58.7 (in at%). Our samples were thus consistently more Mn‐deficient than expected from the nominal chemical formula MnBi_6_Te_10_ of the atomically ordered material (Mn: 5.9, Bi: 35.3; Te: 58.8). Again, this echoes our earlier published single‐crystal structure refinement of Mn_0.73(4)_Bi_6.18(2)_Te_10_ by X‐ray diffraction,^[^ ^31^ ^]^ where we systematically showed that Mn‐substoichiometry is determined by the Mn/Bi intermixing. Both features are also present in Mn_0.85_Bi_2.10_Te_4_^[^ ^32^ ^]^ and Mn_0.75_Bi_4.17_Te_7_.^[^ ^26^ ^]^ To facilitate perception, we denote our samples as MnBi_6_Te_10_ in the following text, keeping in mind that they are in fact substoichiometric.

The present study was performed on four individual Mn‐deficient MnBi_6_Te_10_ crystals (denoted as Sample #1–#4 henceforward; for their chemical compositions (EDX) see Figure S2, Supporting Information). Powder X‐ray diffraction (PXRD) measurements, which required grinding the crystals to a homogeneous powder, were conducted after all other measurements had been finalized, in order to elucidate the underlying intermixing phenomenon. We confirmed that all four samples exhibit the crystal lattice of MnBi_6_Te_10_ with a sequence of one SL and two QLs (Figure 1a) plus notable cation antisite disorder. MnBi_6_Te_10_ constituted the main phase as per Rietveld method and we established a firm link between the Mn content as found by EDX and the underlying crystal lattice of MnBi_6_Te_10_ in our samples.

This approach is exemplified on sample #2 (see Figure 1b and more procedural details in Section SI, Supporting Information). We confirmed that sample #2 was Mn_1 − *x*_Bi_6 + *x*_Te_10_ (*x* ≈ 0.20 − 0.25) which crystallized in the rhombohedral space group $R\bar{3}m$ (No. 166) with the unit cell lattice parameters *a* = 4.36778(8) Å and *c* = 101.8326(6) Å. To stabilize a further Rietveld refinement, the EDX compositions (e.g., Mn_0.76_Bi_6.24_Te_10_ or Mn_0.8_Bi_6.2_Te_10_) were introduced as constraints (see Section SI, Supporting Information). When cation Mn/Bi intermixing was allowed in the refinement, the reliability factors *R*_all_ and *R*_obs_ dropped down significantly, confirming that this phenomenon was undoubtedly present in the structure. Due to very low sample mass (1–2 mg), the acquired powder diffraction data did not allow us to settle in for just one particular intermixing model with a statistically unequivocal quantification. The refined Mn content is also strongly dependent on whether cation vacancies are allowed in the refinement. We opted for a structural solution without voids in the 3*a* and 6*c* positions. Despite the outlined uncertainties, all tested models with various composition constraints have in common that: 1) the Mn:Bi ratio in the 3*a* position in the center of an SL is close to 56:44; 2) the outer cation site of an SL (6*c*) contains up to 2% Mn; 3) the QL always accommodates some Mn (2–7% Mn) in the 6*c* cation sites. The presence of Mn in all cation positions accords with our earlier reported refinement on Mn_0.81_Bi_6.13_Te_10_ single crystals^[^ ^31^ ^]^ and is in contrast to the findings of Klimovskikh et al.^[^ ^23^ ^]^ Such subtle variations in intermixing patterns can dramatically impact the magnetic properties, as witnessed in the next subsection.

### 2.2 Bulk Magnetometry

**Figure 2:**
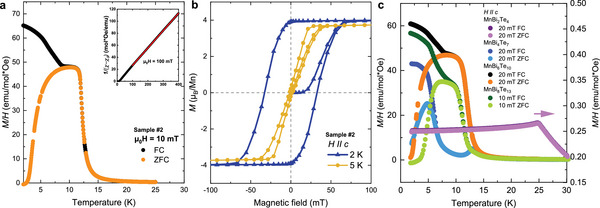
SQUID magnetometry measurements. a) Temperature‐dependent normalized magnetization *M*/*H* of sample #2 with ZFC (orange symbols) and FC (black symbols) protocols in an out‐of‐plane applied magnetic field of 10 mT. The inset shows the inverse magnetic susceptibility in a magnetic field of 100 mT together with a modified Curie–Weiss fit χ(*T*) = χ_0_ + *C*/(*T* − Θ_CW_) of the data above 100 K (red solid line); for details see Experimental Section. b) Field‐dependent magnetization of sample #2 measured in an out‐of‐plane applied magnetic field at *T* = 2  and 5 K. No demagnetization correction was applied, and the magnetization was normalized to the Mn content obtained by EDX. c) Temperature dependence of the normalized magnetization of analogously synthesized samples of the MBT_*n*_ family for (*n* = 0, 1, 2, 3).

**Figure** **2**a shows the field‐cooled (FC) and zero‐field cooled (ZFC) normalized magnetization of sample #2 in an out‐of‐plane magnetic field of 10 mT. A phase transition into a long‐range magnetically ordered state is observed at *T*_c_ = 12.0 K, determined by the inflection point, together with a notable FC/ZFC splitting around 10 K. These observations point toward a ferromagnetic alignment of the Mn spins in our MnBi_6_Te_10_ samples and contrast with the antiferromagnetic transition at *T*_N_ ≈ 11 K so far reported for the nominal MnBi_6_Te_10_ composition.^[^ ^23^, ^25^, ^27^, ^33^, ^34^ ^]^ Our Curie–Weiss analysis in the temperature regime 100–400 K (see inset of Figure 2a and Experimental Section) yields an effective moment of *m*_eff_ = 5.8 ± 0.1 μ_B_/Mn in close agreement with the value *m*_eff_ = 5.67μ_B_ calculated by multiplet ligand‐field theory (MLFT) (Section 2.5). The uniformity of all four MnBi_6_Te_10_ crystals is strongly supported by the nearly identical SQUID magnetometry curves (see Figure S4, Supporting Information), with transition temperatures that vary by only 0.1 K.

The magnetization curves *M*(*H*) in Figure 2b show clear FM loop openings, with a coercive field of μ_0_ *H*_c_ ≈ 32 mT at *T* = 2 K, and a finite remanent moment of $m_{\mathrm{SQ}}^{\mathrm{tot}}=\left(3.9\pm 0.2\right)\mu_{B}/\text{Mn}$ at zero magnetic field. The moment at 0.15 T is $m_{\mathrm{SQ}}^{\mathrm{tot}}=\left(4.2\pm 0.2\right)\mu_{B}/\text{Mn}$.

It is furthermore interesting to compare our results to analogously synthesized samples of the MBT_*n*_ family (Figure 2c). We observe a noteworthy trend as the number of quintuple Bi_2_Te_3_ layers *n* increases: MnBi_2_Te_4_ (*n* = 0) has a clear A‐type AFM structure, whereas MnBi_4_Te_7_ (*n* = 1) exhibits a more complex behavior, in which robust low‐temperature metamagnetic properties are established, which were shown to result from the competition between the uniaxial anisotropy *K* and the still sizable interlayer AFM interaction *J*.^[^ ^21^ ^]^ Finally, in MnBi_6_Te_10_ (*n* = 2), as well as in MnBi_8_Te_13_ (*n* = 3), the FM properties clearly dominate, with FM order at the significant temperatures of *T*_c_ = 12 and 10 K, respectively. Consistent with this observation, the spin‐flop transition found for MnBi_2_Te_4_ and MnBi_4_Te_7_ at fields of 3.5 T^[^ ^32^, ^35^, ^36^ ^]^ and 0.1–0.3 T,^[^ ^23^, ^26^, ^33^, ^37^ ^]^ respectively, is absent in MnBi_6_Te_10_, and a magnetic moment of more than 4μ_B_ is observed already above 80 mT in the latter after a ZFC procedure.

### 2.3 Bulk DFT (GGA+*U*) Calculations

We have first performed fully relativistic DFT calculations based on the Generalized Gradient Approximation (GGA)+*U* ^[^ ^38^ ^]^ for MnBi_6_Te_10_ neglecting the intermixing. For the interaction parameters, we have used the Slater integrals in **Table** **1** for the initial state. The results of total energy calculations for the A‐type AFM configuration favor the out‐of‐plane over the in‐plane magnetization by ≈0.4 meV per Mn. Additional calculations indicate that the A‐type AFM configuration has a lower energy than the FM configuration. However, the small magnitude of the difference, ≈0.04 meV per Mn, naturally suggests that other mechanisms such as Mn/Bi intermixing may well be relevant for the magnetic ground state.

**Table 1:** Slater integrals obtained from DFT and spin–orbit coupling constants in the Hartree–Fock approximation for the Mn^2 +^ ion (in units of eV)

Ion	State	Configuration	$F_{\text{dd}}^{\left(2\right)}$	$F_{\text{dd}}^{\left(4\right)}$	ζ_3d_	$F_{\text{pd}}^{\left(2\right)}$	$G_{\text{pd}}^{\left(1\right)}$	$G_{\text{pd}}^{\left(3\right)}$	ζ_2p_
Mn^2 +^	Initial	2p^6^3d^5^	9.4323	5.8132	0.040				
	Final	2p^5^3d^6^	10.1963	6.2899	0.053	5.3354	3.8379	2.1773	6.846

Taking into account the Mn/Bi intermixing for MnBi_6_Te_10_, with its lattice parameter *c* > 100 Å, would require a prohibitively long computational time. Instead, here we aim to learn the effects of Mn/Bi intermixing on magnetism via the simpler models of MnBi_2_Te_4_ and MnBi_4_Te_7_. The latter case is more representative of MnBi_6_Te_10_, since it contains both SLs and QLs, and is discussed in detail below, while the former is presented in Section SIIIA, Supporting Information. Here, we emphasize one conclusion about MnBi_2_Te_4_: even though its defect‐free form has the strongest AFM coupling between Mn in the consecutive SLs, based on our calculations, intermixing can induce the FM order between the SLs even in this compound (Figure S5, Supporting Information). Hence, the emergence of strong out‐of‐plane FM correlations due to the intermixing is likely to be universally present in the MBT_*n*_ family, including MnBi_6_Te_10_.

**Figure 3:**
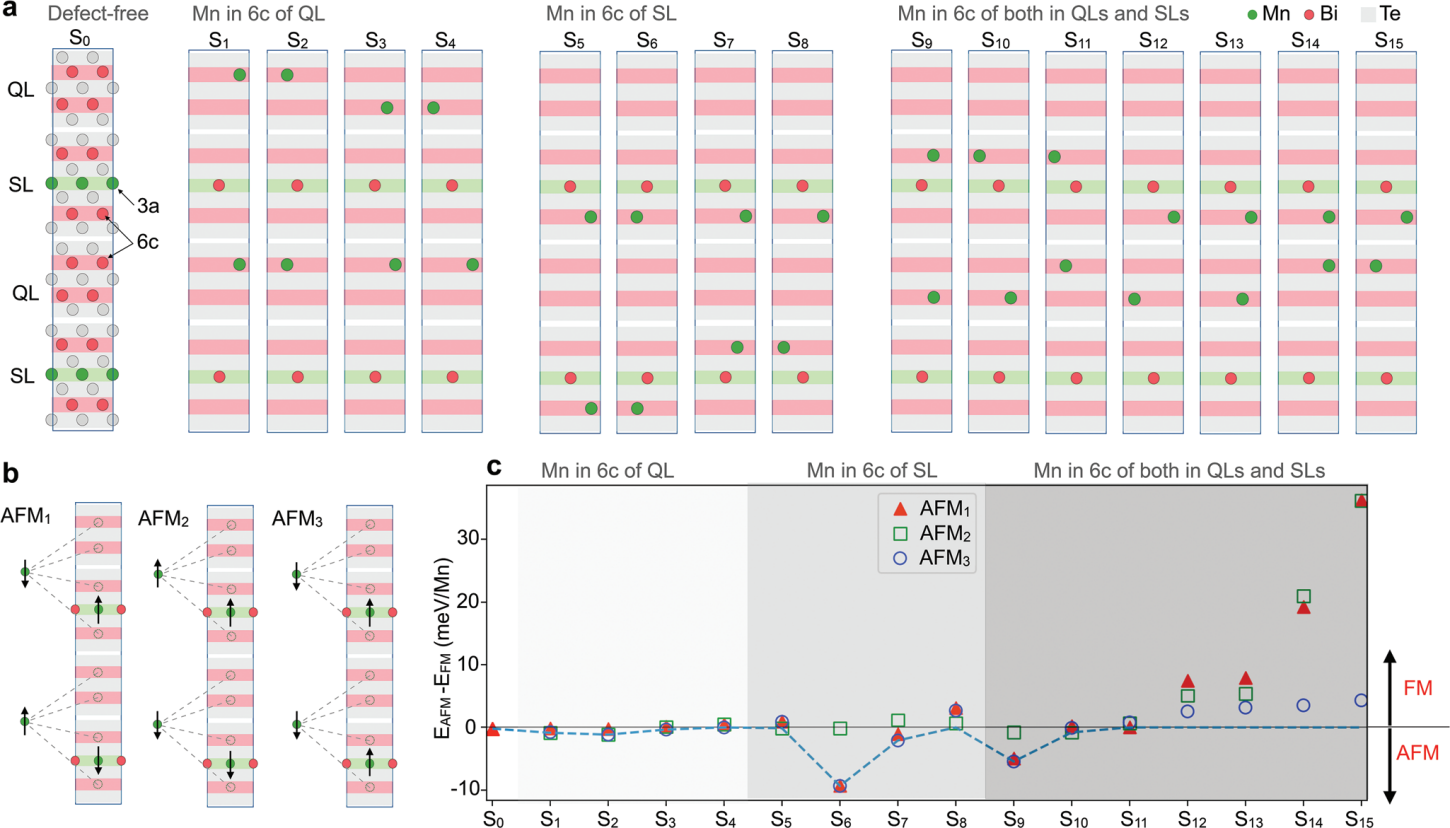
Crystal‐ and magnetic‐structure models and their DFT energies. a) Schematics of the structure models with various Mn/Bi intermixing scenarios. S_0_ is the defect‐free case while the panels from S_1_ to S_15_ visualize the structural differences to S_0_. Models S_1_ to S_4_: antisite Mn (green) is in the 6*c* site of the QLs; models S_5_ to S_8_: antisite Mn is in the 6*c* site of the SLs; models S_9_ to S_15_: antisite Mn is in the 6*c* positions of both QLs and SLs. b) Schematics of the constructed magnetic arrangements. Each model has a distinct order between the Mn magnetic moments in the 3*a* and 6*c* positions which are conditioned by the respective structure model in (a). Ferromagnetic order with an out‐of‐plane orientation of the moments is assumed within each atomic layer. c) Total energy difference between an antiferromagnetic ordering model (1, 2, or 3) of each structure model (S_0_ to S_15_) and the respective fully ferromagnetic configuration, as obtained from the scalar‐relativistic DFT calculations. The dashed line follows the ground state energy, zero corresponding to the FM phase.

As *n* increases, the possibilities for intermixing patterns naturally become larger as antisite Mn atoms can be located in the 6*c* positions (occupied by Bi in the defect‐free case) of both the SLs and the QLs. MnBi_4_Te_7_ provides the minimal framework to explore whether this enlarged configuration space can yield variations in the experimentally observed ground states. We have performed scalar‐relativistic calculations for various intermixing patterns and magnetic orders in a 2 × 1 × 2 supercell of MnBi_4_Te_7_ (**Figure** **3**). In addition to the defect‐free case (S_0_), we construct models (S_1_ to S_15_) with different Mn/Bi antisite defects, all of them globally stoichiometric and having a 50% fraction of Bi atoms in the 3*a* Wyckoff site. Notice that this concentration is close to the outcome of our Rietveld refinements (≈44%). The models differ in the positions occupied by the antisite Mn atoms and can be classified into three categories. In the first category, the antisite Mn atoms reside in the 6*c* position of *only* the QLs (S_1_ to S_4_). Similarly, in the second category antisite Mn occupy the 6*c* positions of *only* the SLs (S_5_ to S_8_). In the third category, the Mn atoms are distributed over the 6*c* positions of *both* the QLs and the SLs (S_9_ to S_15_).

For each structure model, we consider four possible magnetic arrangements: a fully spin‐polarized FM order and three different AFM models sketched in Figure 3b. They all have in common that the Mn moments order FM within any given atomic layer, but vary in the magnetic couplings between the adjacent atomic layers along the stack. In the AFM_1_ and AFM_2_ models, the Mn spins in the two consequent 3*a* positions are oppositely coupled. The coupling between the 3*a* site and all intermixed Mn neighbors in the 6*c* site(s) is either AFM (AFM_1_) or FM (AFM_2_), respectively. The AFM_3_ model realizes parallel spin arrangement in the 3*a* sites, while they couple AFM with the Mn defects in all 6*c* positions.

Figure 3c discerns what is an energetically favorable magnetic arrangement for each considered structure model of MnBi_4_Te_7_ as compared to the fully spin‐polarized FM state. For a given model, if any AFM model obeys *E*_AFM_ − *E*_FM_ < 0, we conclude that antiferromagnetism is preferred. On the other hand, if all AFM models fulfill *E*_AFM_ − *E*_FM_ > 0, we define the fully FM phase as the ground state.

A clear trend in the magnetic order as a function of the underlying Mn/Bi intermixing pattern can be established. All but one models of the first two categories, where the intermixed Mn cations occupy *either* the SLs *or* the QLs, show an AFM configuration as the lower energy state. This preference reverts markedly if the antisite Mn distributes over the 6*c* positions of *both* the QLs and the SLs: five out of the seven constructed structure configurations of this category prefer the FM phase. A closer look reveals that the preference for the FM state is particularly prominent in those structure models (S_12_ to S_15_), in which the Mn cations occupy the nearby 6*c* positions not separated by the 3*a* positions—namely, when continuous magnetic exchange pathways exist between the antisite Mn ions.

These results establish a strong correlation between the magnetic structure and the Mn distribution along the stacking direction. When an antisite Mn is located only in one of the two 6*c* positions of the QLs—a situation experimentally found in ref. [23]—our calculations suggest the prevalence of an AFM order. When Mn distributes both in the 6*c* of the QLs and SLs, which is the case of our samples according to our structure refinements, our calculations identify the FM phase as the ground state.

### 2.4 X‐Ray Absorption Spectroscopy and XMCD Data

To study the surface magnetic properties, we have performed X‐ray absorption spectroscopy (XAS) measurements in the total electron yield (TEY) mode, which is element specific and has a probing depth on the nanometer scale. Measurements at the Bi *N*_4, 5_ edges (Figure S7b, Supporting Information) exhibit no XMCD. More interestingly, there is no XMCD at the Te *M*_4, 5_ edges either (Figure S7a, Supporting Information), which is in contrast to results in the closely related V‐ and Cr‐doped (Bi,Sb)_2_Te_3_,^[^ ^39^, ^40^, ^41^, ^42^ ^]^ and which might indicate differences in the magnetic interactions of both compounds.

**Figure 4:**
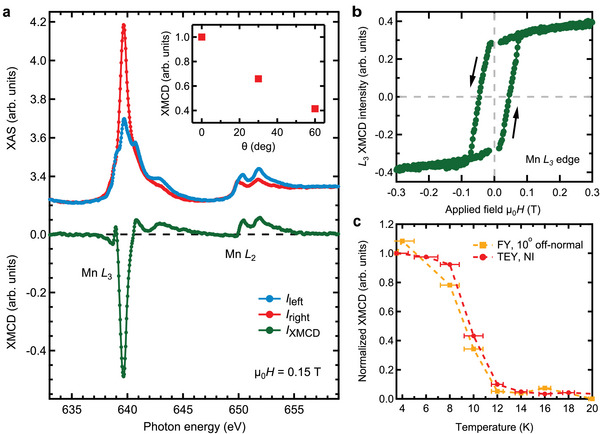
X‐ray spectroscopy data. a) Mn *L*_2, 3_ edge XAS data for sample #4 obtained with left (*I*_left_, blue) and right (*I*_right_, red) circularly polarized light in normal incidence at *T* ≈ 3.5 K in a magnetic field of 0.15 T. The corresponding XMCD signal *I*_XMCD_ = *I*_left_ − *I*_right_ is plotted below in green. The inset shows the angular dependence of the normalized remanent XMCD signal. b) Magnetization curve of sample #4 (*I*_right_ − *I*_left_) at *T* ≈ 3.5 K, obtained as the Mn *L*_3_ edge XMCD signal normalized by the XAS signal. c) Temperature dependence of the remanent XMCD signal for sample #2 at the Mn *L*_3_ edge measured at normal incidence in TEY mode (red) and 10° off normal incidence in FY mode (orange).

Next we focus on the Mn *L*_2, 3_ edge. Due to the shallow escape depth, the topmost SL contributes the most to the signal. However, even for SL termination, the FM Mn sheet is buried about 0.55 nm below the surface, and significantly more for QL termination. Therefore, probing depth effects have to be considered, when interpreting the ordered magnetic moments obtained with XMCD (see Sections 2.6 and 3). **Figure** **4**a compares XAS spectra measured with X‐rays of opposite circular polarization at *T* ≈ 3.5 K and a magnetic field of $\mu_{0}H=0.15\;T$ along the surface normal; the bottom green line showcases the substantial XMCD signal. In the inset we show that the peak remanent XMCD signal scales inversely with θ, where θ is the angle between the magnetization direction and the X‐ray beam. This decline of XMCD is a strong indication of an out‐of‐plane easy axis for the Mn moments.

In Figure 4b we show the magnetization obtained by measuring the peak *L*_3_ XMCD signal at *T* ≈ 3.5 K within a field range of ±0.3 T. It exhibits a substantial remanence at μ_0_ *H* = 0 T, in sharp contrast to MnBi_2_Te_4_, which exhibits no remanent magnetization, and MnBi_4_Te_7_, which has a smaller remanence‐to‐saturation ratio.^[^ ^26^ ^]^ Furthermore, we observe a coercive field of μ_0_ *H*_c_ = 45 mT. We caution against overinterpreting the similarity of this *H*_c_ with the bulk one: First, the data were measured at somewhat different temperatures, which has an effect on *H*_c_ (Figure 2). Second, different ramping speeds were used, which, too, has an effect on *H*_c_ for magnetic TIs.^[^ ^43^ ^]^ In addition, the hysteretic behavior of surface and bulk might be intrinsically different.

Finally, in Figure 4c we compare the *T*‐dependent remanent peak *L*_3_ XMCD signal measured with surface sensitive TEY with the one measured with bulk sensitive total fluorescence yield (FY). Within the precision allowed by the *T* increments of 2 K, the transition temperatures at surface and bulk are consistent. We remark that the transition behavior as observed with SQUID and XMCD could differ somewhat due to the different measurement protocols: For XMCD, each point in Figure 4c was obtained after driving to μ_0_ *H* = 3 T and back to remanence. In contrast, in SQUID measurements a conventional FC protocol at 10 mT was used.

### 2.5 MLFT Calculations

The line shapes of the XAS and XMCD spectra contain important physical information, such as the d‐electron configuration, including the local magnetic moments. Therefore, we have modeled our experimental data by MLFT. In our approach^[^ ^39^, ^44^, ^45^, ^46^ ^]^ (Experimental Section), rather than relying on oversimplified approximations, we adjust most of the MLFT parameters to the data and obtain 10*Dq* = 0.06, 10*DqL* = 2*T*_pp_ = 1.9, Δ = 1.1, *U*_dd_ = 4.0, *U*_pd_ = 5.0, $V_{e_{g}}=1.3$ and $V_{t_{2g}}=0.65$ (all in units of eV). For the SO coupling constants, we use the Hartree‐Fock values,^[^ ^47^ ^]^ whereas the Slater integrals are calculated based on DFT in the local density approximation (LDA, Table 1).

**Figure 5:**
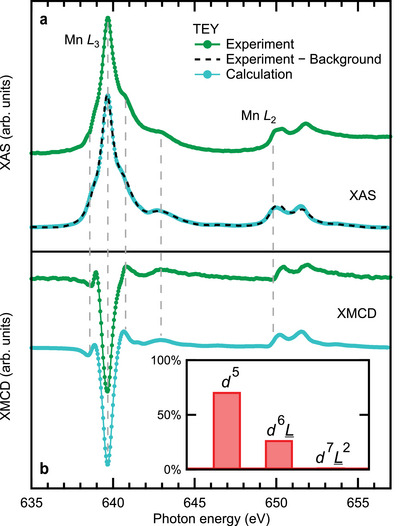
MLFT analysis. a) Background‐corrected polarization‐averaged experimental XAS spectrum (dashed line) together with a calculated MLFT spectrum (blue). The original, uncorrected data is shown above (green line). b) Corresponding experimental and calculated XMCD spectra. The inset shows the contributions of different electronic configurations to the ground state. The vertical dashed lines highlight the positions of particular features of the spectra.

The calculated spectra (**Figure** **5**) show an excellent agreement with the experimental data, most notably for the XMCD, reproducing all the multiplet features and their relative energy positions. Whereas the nominal Mn^2 +^ d^5^ configuration (^6^ *S*_5/2_) dominates with 71%, there is significant charge transfer from the Te ligands, resulting in a 27% contribution of d${}^{6}\underline{L}$ to the ground state (d${}^{7}{\underline{L}}^{2}$ contributes negligibly, see the inset in Figure 5b). This hints toward a considerable hybridization between Mn d and ligand p orbitals. The resulting 3d electron filling is *n*_d_ = 5.31, corresponding to an effective 1.69 + valence. We obtain $m_{\mathrm{eff}}^{\mathrm{spin}}=\sqrt{\left\langle m^{2}\right\rangle }=5.67\mu_{B}$ for the local effective moment, as well as $m^{\mathrm{spin}}=g_{s}\left\langle S_{z}\right\rangle =4.68\mu_{B}$ and *m* ^orb^ = *g*_*l*_〈*L*_*z*_〉 = 0.008μ_B_ for the maximal *z*‐projections of the spin and orbital moments, respectively.

Finally, we observe that the electron filling and the magnetic moments resulting from our MLFT analysis are in excellent agreement with those calculated based on the DFT‐GGA+U calculations in Section 2.3. We obtain *n*_d_ = 5.3 and *m* ^spin^ = 4.7μ_B_ using the same interaction parameters as in MLFT. These results are also in good agreement with the bulk magnetometry data (see Section 2.2 and Figure 2), as well as with published neutron diffraction data.^[^ ^34^, ^48^ ^]^

### 2.6 XMCD Sum Rule and Peak Asymmetry Analysis

**Figure 6:**
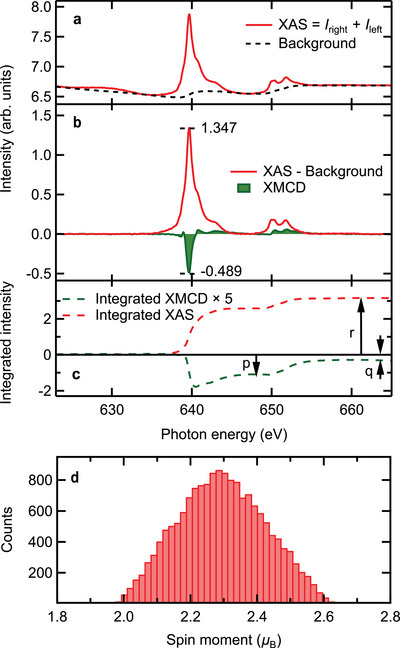
Sum rule and peak asymmetry analysis of data measured on sample #4 at *T* ≈ 3.5 K and $\mu_{0}H=0.15\;T$. a) Polarization‐averaged XAS intensity *I* (red line) together with the background (dashed line). b) XAS spectrum after background correction (see also Supporting Information Section SV‐A), together with the XMCD signal *I*_XMCD_ (filled green curve). The peak intensities necessary for the asymmetry analysis are marked. c) Integrated intensities of the XAS and the XMCD (multiplied by 5) spectra. The integrals *p*, *q*, and *r* necessary for the sum rules are indicated with arrows. d) Distribution of $m_{\mathrm{XM}}^{\mathrm{spin}}$ obtained by randomly varying the sum rule parameters within reasonable error margins, but not considering the uncertainty in the background choice (Section 2.6 and Section SV, Supporting Information).

The MLFT analysis yields the *local* magnetic moments based on the spectral *shape*. The sum rules, in turn, relate the integrated Mn *L*_2, 3_ XMCD and X‐ray absorption spectral *intensity* to the *long‐range ordered* orbital and spin magnetic moments near the surface^[^ ^49^, ^50^, ^51^ ^]^ (Section 4 and Section SV‐A, Supporting Information). We show their application to data for sample #4 in **Figure** **6**. After a background correction (Figure 6a), we obtain the XAS and XMCD data (Figure 6b), from which we calculate the integrals *p*, *q*, and *r* required for the sum rule analysis (Figure 6c). Finally, in Figure 6d we show the distribution of the resulting values for $m_{\mathrm{XM}}^{\mathrm{spin}}$ obtained by applying the analysis 16384 times while randomly varying the sum rule parameters within reasonable error margins. Also taking into account some ambiguity in the choice of the XAS background due to the rather featureless but intense tails of the preceding Te *M*_4, 5_ edges allows us to estimate the errors (Section SV, Supporting Information). We obtain $m_{\mathrm{XM}}^{\mathrm{spin}}=2.3\pm 0.25$, $m_{\mathrm{XM}}^{\mathrm{orb}}=0.1\pm 0.15$ and $m_{\mathrm{XM}}^{\mathrm{tot}}=2.4\pm 0.3$ (in μ_B_/Mn, **Table** **2**). The same analysis for sample #1 yields $m_{\mathrm{XM}}^{\mathrm{tot}}=\left(2.2\pm 0.35\right)\mu_{B}/\text{Mn}$, which is compatible with sample #4 within the error.

**Table 2:** Comparison of the magnetic moments obtained from analysis of surface sensitive XAS and XMCD data measured at *T* ≈ 3.5 K in a 0.15 T field with bulk‐sensitive SQUID magnetometry results at *T* = 2 K and the same field (in units of $\mu_{B}$/Mn). The error bars are explained in Supporting Information sec V

Sum rules:	$m_{\mathrm{XM}}^{\mathrm{spin}}$	2.3 ± 0.25
	$m_{\mathrm{XM}}^{\mathrm{orb}}$	0.1 ± 0.15
	$m_{\mathrm{XM}}^{\mathrm{tot}}$	2.4 ± 0.30
Asymmetry:	$m_{\mathrm{XM}}^{\mathrm{spin}}$	2.55 ± 0.25
SQUID:	$m_{\mathrm{SQ}}^{\mathrm{tot}}$	4.2 ± 0.2

An alternative way to obtain $m_{\mathrm{XM}}^{\mathrm{spin}}$ is to analyze the XMCD *L*_3_ peak asymmetry,^[^ ^52^, ^53^, ^54^ ^]^ which avoids the problems arising from uncertainty in *p* due to the overlap of the *L*_3_ and *L*_2_ peaks (Section SV‐B, Supporting Information). We obtain $m_{\mathrm{XM}}^{\mathrm{spin}}=\left(2.55\pm 0.25\right)\mu_{B}/\text{Mn}$, which is about 10% larger than the sum rule result.

Table 2 also shows that the orbital moment is negligible within the error, as expected for a predominant d^5^ configuration (Section 2.5). The total moment $m_{\mathrm{XM}}^{\mathrm{tot}}$ obtained with surface sensitive XMCD is reduced by about 40% in comparison to the one obtained with bulk sensitive SQUID magnetometry. Increasing the field to 6 T brings the moment $m_{\mathrm{XM}}^{\mathrm{tot}}$ to 3.9 μ_B_, that is, closer to $m_{\mathrm{SQ}}^{\mathrm{tot}}=4.2\;\mu_{B}$ and to the theoretical maximal moment (Section 2.5).

It is important to keep in mind that the indicated errors of the XMCD results only take into account statistical fitting and background estimate effects. However, the shallow probing depth can further bias the outcome (Section 2.4 and ref. [55]): It is reasonable to expect that the QLs and the outer (6*c*) positions of the SLs might contain slightly canted Mn, as well as a few percent of paramagnetic and possibly even AFM (with respect to the 3*a* positions) Mn, see Section 2.1, Section 3 and Figure 1. Already for SL surface termination, the FM ordered Mn sheet (3*a* positions) is buried about 0.55 nm below the surface. At the same time, there is Mn in 6*c* positions closer to the surface, both in the very same SL and in QLs, which can terminate the surface in different parts of the sample. Therefore, already for a mean probing depth (MPD) of about 1 nm, the contribution of the FM Mn would be notably suppressed. MPD values between 1 and 2.5 nm have been reported for this photon energy range.^[^ ^56^, ^57^ ^]^ Given the presence of the heavy elements Te and Bi, which might even further attenuate the escaping electrons, an MPD close to 1 nm is not unrealistic and the involvement of probing depth effects is well conceivable.

## 3 Discussion

The major finding of our study is that the surface of MnBi_6_Te_10_ exhibits FM properties comparable to its bulk, with a robust FM subsystem in the topmost septuple layer, which can interact with the topological surface states. Indeed, recent ARPES reports suggest the opening of an exchange gap of about 15 meV.^[^ ^58^ ^]^

As outlined in Section 2.6, probing‐depth effects can at least partially explain the 40% reduction of the XMCD‐derived remanent moment as compared to the SQUID‐derived bulk moment. Additional surface effects might influence magnetism and therefore warrant consideration. First of all, the incomplete out‐of‐plane coordination by magnetic neighbors of the topmost SL suppresses the out‐of‐plane magnetic interactions and interrupts the exchange paths of the antisite Mn ions. However, since these interlayer interactions are weak (Section 2.3), additional theoretical scrutiny would be required to elucidate, what role their further suppression might play. Second, a competition between the demagnetizing field and the crystalline anisotropy might result in a canting and a suppression of the XMCD signal—an effect which must, however, be small due to our finding of a strong out‐of‐plane anisotropy, see Section 2.4 and the inset of Figure 4a. Third, a combined study involving DFT and XMCD suggests that TSS couple to magnetic atoms such as Co and Mn at the surface of Bi_2_Te_3_, contributing to their interaction by a RKKY‐like mechanism:^[^ ^59^ ^]^ Due to their highly localized nature, electrons in the TSS interact more strongly with magnetic moments than electrons in the bulk. This might contribute to the differences between the magnetic properties at the surface and in the bulk. Our results encourage similar calculations for MnBi_6_Te_10_. Finally, the interaction with the TSS might also result in a slight helical canting away from the out‐of‐plane orientation, driven by Dzyaloshinskii–Moriya interactions.^[^ ^60^, ^61^ ^]^ Again, due to the strong out‐of‐plane anisotropy we experimentally observe, this contribution would be small.

We now discuss the mechanism inducing the crossover from a pronounced AFM toward an FM order as the number *n* of the QLs in the MBT_*n*_ stacking sequence increases (Section 2.2 and Figure 2). Our calculations for the ordered MnBi_6_Te_10_ (Section 2.3) yield an—albeit small—AFM coupling. Although the increasing *K*/2*J* ratio with increasing *n* ^[^ ^21^ ^]^ certainly helps to stabilize the FM order for *n* ⩾ *n*_FM_ = 2 in our samples (see Section 2.2 and Figure 2c), the fact that previous studies reported *n*_FM_ = 3^[^ ^22^, ^23^, ^24^, ^25^ ^]^ hints at an additional phenomenon being involved. In Sections 2.1 and 2.3 we have established the presence and the role of Mn/Bi antisite defects that can drive enhanced FM properties. As our numerical modeling has shown both for the MBT_0_ with the strongest interlayer AFM coupling and for the MBT_1_ with a periodic alternation of SLs and QLs, the motif of an intermixing pattern determines whether ferro‐ or antiferromagnetism is preferred. Hence, the observed magnetic properties of our MnBi_6_Te_10_ samples likely originate in a prevalence of intermixing patterns that favor the FM order.

In this context, a comparison between the MBT_*n*_ series and the analogous Sb‐based family (MnSb_2_Te_4_)(Sb_2_Te_3_)_*n*_ (MST_*n*_) becomes relevant. The FM order is more dominant even for *n* = 0 in MST_*n*_ and MBST_*n*_, and, as widely accepted by now, is driven by the Mn/Sb intermixing.^[^ ^62^, ^63^, ^64^, ^65^ ^]^ This phenomenon is much stronger in MST_*n*_ than in MBT_*n*_ since it is facilitated by closer atomic radii of Mn and Sb. On the other hand, the impact of the intermixing‐induced FM state in MnSb_2_Te_4_ on its band topology is still under ongoing debate.^[^ ^9^, ^62^, ^63^, ^66^, ^67^, ^68^, ^69^, ^70^ ^]^ Intrinsic p‐type doping in MST_0_ hampers clear‐cut spectroscopic observations of the possible surface states and, thus, an ultimate conclusion about its topological nature. Furthermore, QAHE realizations in the MST_*n*_ have not been reported. In fact, ref. [63] argues the importance of further studies on how intermixing impacts bulk and surface magnetism in the established topological MBT_*n*_ materials, but focuses on the MST_0_ instead, because the necessary intermixings were not accessible by the bismuth analog at that time. So far intermixing in the MBT_*n*_ has been discussed mostly in the terms of its influence on the Dirac‐point gap,^[^ ^71^ ^]^ while the consequences for the magnetism are quite unclear. En route to understanding the broader role of intermixing, a recent study reveals its crucial influence on the magnetic coupling in MnBi_2_Te_4_,^[^ ^72^ ^]^ and our current work pinpoints the particular antisite defects that enhance (or suppress) the local FM coupling in the MBT_*n*_ series.

We have established that the FM properties of our crystals are conditioned by the underlying cation intermixing. It is instructive to examine whether this relationship holds true for the other published works. Whereas Mn deficiency in MnBi_6_Te_10_ is often found by X‐ray spectroscopy,^[^ ^22^, ^24^, ^25^ ^]^ the related intermixing has been scrutinized only in ref. [23]. On the one hand, their and our samples have such commonalities as the presence of Mn/Bi intermixing, the absence of cation vacancies, and a strongly mixed occupancy on the 3*a* site. On the other hand, there are also substantial differences: The mixed 3*a* occupancy is more pronounced in our sample, in which we find 56% Mn (and 44% Bi), than in the sample studied in ref. [23], which has 83% Mn (and 17 % Bi). Most importantly, the Mn distribution over the 6*c* positions is distinctly different: We observe a higher Mn concentration in both 6*c* sites of the QLs, that is, up to Bi_1.86_Mn_0.14_Te_3_ versus Bi_1.92_Mn_0.08_Te_3_ in ref. [23], and up to 2% Mn in the outer positions of the SL that are reported defect‐free in ref. [23]. In general, the 3*a* site in our crystals is more Mn‐depleted, so that these “stray” Mn atoms, which find no space on the 3*a* site, disperse over the entire layered stack by occupying 6*c* sites. In accordance with our theoretical deliberations in Section 2.3 (models S_1_ to S_4_), the less pronounced intermixing and the presence of swapped Mn only in one of the two 6*c* sites of the QLs in the samples of ref. [23] accords with them featuring an AFM ground state.

The question of why intermixing takes place and which kind of defects are more likely to occur is evidently very relevant and, at the same time, a complex one. First, recent literature has shown that antisite cationic defects have the lowest formation energy and are energetically favorable to form in both MnBi_2_Te_4_ and MnBi_4_Te_7_.^[^ ^73^ ^]^ It has been argued that such defects provide an effective way to release a lattice strain effect which occurs within the septuple layer of MnBi_2_Te_4_ due to a mismatch between the MnTe and Bi_2_Te_3_ structure fragments. Second, as argued below, variations in the intermixing patterns of MnBi_6_Te_10_ samples produced by different groups may stem from subtle differences in the synthetic procedures, pointing to the relevance of finite temperature effects for the relative stability of different defects. This would be of no surprise since these compounds are formed at elevated temperatures and are metastable at room temperature as we have previously shown.^[^ ^31^, ^32^ ^]^

Comparing our growth conditions (see Section 4) for MnBi_6_Te_10_ to those of refs. [23, 33, 34] reveal differences in dwelling times, the starting and quenching temperatures, and the composition of a melt, which may well account for the various intermixing patterns. In general, we observe reproducible Mn concentrations and magnetic behavior in our MBT_2_ crystals (Figure 2 and Figure S4, Supporting Information) for an applied tempering profile,^[^ ^31^, ^74^ ^]^ suggesting that the cation intermixing is a temperature‐regulated phenomenon. We do not argue that this process is fully governed by the thermodynamics, since crystallization of the MBT_*n*_ from a heterogeneous melt is strongly kinetics‐driven. Yet it seems plausible, that the resulting intermixing pattern is governed by a given synthetic protocol. Strong correlations between the synthesis temperatures and the resultant cation disorder and magnetic order have been, by now, undoubtedly established at least for MnSb_2_Te_4_.^[^ ^62^, ^74^, ^75^ ^]^ Since the Bi‐analogs have limited thermodynamic stability^[^ ^31^, ^32^ ^]^ and, thus, offer very narrow growth temperature windows, their degrees of intermixing appear to be far less dramatic than in MnSb_2_Te_4_ and, therefore, more challenging to trace experimentally. Less substitutional disorder than in the MST_*n*_ (on average) may be a blessing when it comes to optimizing an MBT_*n*_ material's system for the QAHE device fabrication.^[^ ^29^ ^]^

In summary, the prominent ferromagnetic characteristics of our sample, with a rather large *T*_c_, and a substantial ordered, out‐of‐plane moment both in the bulk and at the surface, categorizes MnBi_6_Te_10_ as a particularly interesting candidate for the realization of a high‐temperature QAH material.^[^ ^20^, ^25^, ^29^, ^37^ ^]^ Moreover, a monolayer of ferromagnetic MnBi_6_Te_10_ appears as a perspective candidate for magnetic extension^[^ ^30^, ^76^ ^]^ and proximity setups, since an FM MnBi_6_Te_10_ slab was predicted to exhibit QAHE.^[^ ^77^ ^]^

## 4 Experimental Section

### Crystal Growth and Characterization

Pre‐synthesized, phase‐pure MnTe and Bi_2_Te_3_ powders were mixed in a ratio 0.85 : 2 at%, pelletized and placed in an evacuated quartz tube. This was inserted at *T* = 923 K into a preheated two‐zone tube furnace with temperature control via external thermocouples (Reetz GmbH). The ampule was subsequently cooled down to 858 K at a rate of 1 K h^−1^, tempered for 14 days and then quenched in water. Platelet‐like MnBi_6_Te_10_ crystals (lateral size up to 1 mm) were mechanically separated from the obtained ingot.

Powder X‐ray diffraction data were collected on an X'Pert Pro diffractometer (PANalytical) with Bragg–Brentano geometry (featuring variable divergence slits) operating with a curved Ge(111) monochromator and Cu‐Kα_1_ radiation (λ = 154.056 pm). The phase composition of the polycrystalline ingot and individual crystals was estimated by Le Bail or Rietveld methods in JANA2006.^[^ ^78^ ^]^ The preferred orientation of the crystallites was described by March–Dollase corrections, the roughness for the Bragg–Brentano geometry was accounted for by the Suorti method.

Scanning electron microscopy (SEM) was performed using a SU8020 (Hitachi) equipped with a X‐MaxN (Oxford) Silicon Drift Detector (SDD) at *U*_a_ = 2 − 5 kV. The composition of selected single crystals was determined by semi‐quantitative energy dispersive X‐ray analysis at 20 kV acceleration voltage.

### Bulk Magnetometry Measurements

Field and temperature dependent magnetization studies were performed using a quantum design SQUID magnetometer equipped with a vibrating sample magnetometer (VSM) option (MPMS3). The magnetization data on samples #1–#4 were normalized to the real compositions determined via EDX. To obtain the absolute magnetization *M* per Mn atom, a precise knowledge of the sample mass is important. Samples #1 and #4 have ≈10 times smaller mass than sample #2, increasing the error of *M*. Nevertheless, the data for all four samples agreed well with each other (Figure S4, Supporting Information). Furthermore, here the projection of the total magnetic moment onto the *z*‐axis (H||z), which was the quantity obtained from the SQUID magnetometry measurements, is referred to as $m_{\mathrm{SQ}}^{\mathrm{tot}}$.

A setup made of two half‐cylindrical quartz rods fixed with a small quantity of GE varnish to the main quartz VSM sample holder was designed to ensure an alignment of the crystals such that the external magnetic field was applied perpendicular to the crystal surface. Note that this setup, however, resulted in a rather temperature‐independent (at not too low temperature) but non‐negligible background contribution to the magnetic susceptibility, hindering a reliable extraction of the Curie–Weiss constant θ_CW_ and the temperature independent susceptibility χ_0_ for the low‐mass samples MnBi_6_Te_10_.

### Bulk DFT (GGA+*U*) Calculations

Fully relativistic DFT calculations based on the GGA + *U* were performed with the parametrization of Perdew, Burke, and Ernzerf,^[^ ^38^ ^]^ using the full localized limit for the double‐counting correction with *U* = *U*_dd_ (the latter as obtained in Section 2.5) and $J=(F_{\text{dd}}^{\left(2\right)}+F_{\text{dd}}^{\left(4\right)})/14$, with $F_{\text{dd}}^{\left(2\right)}$ and $F_{\text{dd}}^{\left(4\right)}$ the Slater integrals for the initial states presented in Table 1. The spin–orbit coupling was included in the four‐component formalism as implemented in FPLO. The total energy difference between the FM and A‐type AFM configurations was computed using a linear tetrahedron method for Brillouin zone integrations. For MnBi_6_Te_10_, a mesh of the Brillouin zone having 14 × 14 × 14 subdivisions was used. The magnetic anisotropy energy was calculated in the AFM state based on a mesh having 10 × 10 × 10 subdivisions. The Mn 3d occupancy and the spin projection presented in the main text correspond to the gross projections. For the calculations of MnBi_4_Te_7_ based on a 2 × 1 × 2 supercell, a mesh with 6 × 12 × 2 subdivisions was used.

### XAS and XMCD Measurements

The XAS and XMCD measurements were performed using the high‐field cryomagnet end station HECTOR of the BOREAS beamline at the ALBA synchrotron radiation facility^[^ ^79^ ^]^ and at the high‐field diffractometer at the UE46 PGM‐1 beamline, BESSY II.^[^ ^80^ ^]^ The single crystals were glued with conducting silver epoxy onto Cu sample plates and mounted on the cold finger of a helium flow cryostat. Prior to the measurements, each sample was mechanically cleaved in the fast‐entry chamber at a pressure of ≈10^−9^ mbar to expose a pristine surface. The sample was then transferred into the spectroscopy chamber with a pressure in the 10^−11^–10^−10^ mbar range.

The measurements were carried out in the TEY or FY mode at magnetic fields of up to 6 T and at various temperatures in the 3.5–35 K range. The temperature was calibrated with a thermal sensor mounted at the sample position before the experiment. Especially below about 5 K, the actual sample temperature crucially depends on the thermal contact, increasing its error as compared to higher temperatures. The spectral intensity was normalized by the incoming photon intensity (*I*_0_). Circularly polarized light was used at both beamlines. The area probed by the beam at both facilities (about $120\times 80\;\mu m^{2}$) was much smaller than the sample size.

The raw XAS spectra were scaled with respect to each other to have the same intensity at energies far from the resonances to obtain *I*_left_ and *I*_right_. The XMCD signal is defined as *I*_XMCD_ = *I*_left_ − *I*_right_. The average, not background corrected XAS is *I* = (*I*_left_ + *I*_right_)/2. To cancel out any experimental drifts, for each data set eight spectra were measured in a row by altering the X‐ray polarization. Finally, the magnetic moments measured with XMCD are marked with the subscript XM, for example, $m_{\mathrm{XM}}^{\mathrm{tot}}$.

### MLFT Calculations

As a starting point to obtain input parameters for the MLFT modeling, self‐consistent DFT in the linear density approximation is sufficient, which was performed using the FPLO package.^[^ ^81^ ^]^ The Brillouin zone was sampled by a 2 × 2 × 2 *k*‐point mesh. The exchange‐correlation potential was treated in LDA, with the scalar relativistic functional according to ref. [82]. The experimental crystal structure from ref. [31] was used: rhombohedral space group $R\bar{3}m$ (166), *a* = 4.37 Å and *c* = 101.83 Å, slightly distorted octahedral Mn coordination with Mn−Te bond length of 3.00 Å (*C*_3*v*_ crystal field symmetry) was used. Wannier orbitals were obtained as input for MLFT by downfolding to a basis set of Mn 3d, Te 5p, and Bi 6p orbitals in an energy window from −6 to 3 eV including an exponential decaying tail with a decay of 1 eV at the boundaries of the selected energy range.

The MLFT calculations were performed using the Quanty package^[^ ^44^, ^83^, ^84^ ^]^ within the CI scheme considering the nominal 2p^6^3d^5^ (Mn^2 +^) configuration and two further charge‐transfer states $d^{6}\underline{L}$ and $d^{7}{\underline{L}}^{2}$. The spectral contributions from the split ground‐state terms were weighted by a Boltzmann factor for *T* = 2 K. The mean‐field effective potential was modeled by an exchange field estimated from the *T*_c_ of 12 K. Instrumental and lifetime effects were taken into account by a Gaussian broadening of 0.35 eV (FWHM) and an *E*‐dependent Lorentzian profile of 0.15 − 0.35 eV (FWHM).

The Slater integrals for the MLFT calculations were obtained by DFT, where $F_{\text{dd}}^{\left(2\right)}$ and $F_{\text{dd}}^{\left(4\right)}$ were scaled up by 8% for the final state, improving the agreement to experiment. SO coupling constants were kept to the Hartree‐Fock values.^[^ ^47^ ^]^ $\Delta =E\left(d^{n+1}\underline{L}\right)-E\left(d^{n}\right)$, *U*_dd_ and *U*_pd_ were directly fitted to the experimental spectra, keeping *U*_dd_/*U*_pd_ = 0.8.^[^ ^85^, ^86^, ^87^, ^88^ ^]^ Experiments involving charge‐neutral excitations such as XAS are only weakly sensitive to Δ, *U*_dd_, and *U*_pd_. In this particular case XAS and XMCD spectra were fitted simultaneously, which substantially mitigated these kind of problems. The results were in good agreement with values reported for (Ga,Mn)As^[^ ^89^, ^90^, ^91^, ^92^, ^93^ ^]^ and Mn‐doped Bi_2_Se_3_^[^ ^55^ ^]^ and Bi_2_Te_3_.^[^ ^94^ ^]^ The other MLFT input parameters were estimated from DFT, and their values were subsequently adjusted to reproduce the experimental spectra. To simplify the calculation, instead of the trigonal *C*_3*v*_ the work was done in *O*_*h*_ symmetry, with the *C*_4_ octahedral axes along the Mn−Te bonds, which had a negligible impact: The simplification here neglected the splitting of the *t*_2g_ orbitals, which is tiny compared to 10*Dq* < 100 meV, which in turn is smaller than the experimental resolution.

### XMCD Sum Rule and Peak Asymmetry Analysis

The XMCD sum rules yield:

(1) $$m_{\mathrm{XM}}^{\mathrm{orb}}=-\frac{4}{3}\frac{q}{r}\left(10-n_{d}\right)$$

(2) $$m_{\mathrm{XM}}^{\mathrm{spin}}=-\frac{6p-4q}{r}\left(10-n_{d}\right)C+7\left\langle T_{z}\right\rangle$$

where *p* and *q* are the XAS intensity differences (*I*_left_ − *I*_right_) integrated over the *L*_3_ edge and the entire *L*_2, 3_ region, respectively (Figure 6). The XAS intensity *I*, after background correction (Section SV‐A, Supporting Information), is integrated over *L*_2, 3_ to yield *r*. 〈*T*_*z*_〉 is the expectation value of the intra‐atomic magnetic dipole operator which is −0.0002ℏ and hence negligible (Section SV‐A, Supporting Information). For *n*_*d*_ the MLFT value of 5.31 (Section 2.5) was used. Finally, *C* is a correction factor, which takes into account the considerable overlap of the *L*_3_ and *L*_2_ contributions for light transition metals. A value of *C* = 1.4 (Section SV‐A, Supporting Information) was used. To circumvent the difficulties related to this overlap, $m_{\mathrm{XM}}^{\mathrm{spin}}$ was also obtained by a comparison of the experimental XMCD asymmetry at the *L*_3_ peak to the theoretical one calculated from MLFT spectra of comparable line width.^[^ ^52^, ^53^, ^54^ ^]^

## Conflict of Interest

The authors declare no conflict of interest.

## Author Contributions

L.F., E.K., and A.I. conducted and analyzed the experimental work related to crystal growth, XRD, and EDX. B.R., L.T.C., and A.U.B.W. performed and analyzed bulk magnetometry measurements. J.I.F. and J.v.d.B. performed and analyzed density‐functional calculations aimed to understand the effects of intermixing. A.T., V.B.Z., T.R.F.P., P.G., M.V., and E.W. performed the XAS measurements. A.T. and V.B.Z. analyzed the XAS data with input and support from T.R.F.P., P.K., S.H., H.B., R.J.G., M.W.H., and V.H. V.B.Z., A.I., and V.H. conceived and supervised the project with input and support from F.R., J.v.d.B., B.B., and A.U.B.W. All authors contributed to the discussion and writing of the manuscript.

## Supporting information

Supporting Information

## Data Availability

The data that support the findings of this study are available from the corresponding author upon reasonable request.
