# Supplementary material for: Intermixing‐Driven Surface and Bulk Ferromagnetism in the Quantum Anomalous Hall Candidate MnBi6Te10
Source: Adv Sci (Weinh). 2023 Feb 17;10(10):2203239. doi: 10.1002/advs.202203239 (PMC10074120; doi:10.1002/advs.202203239)
Supplement: Supplementary file 1 — Supporting Information [file ADVS-10-2203239-s001.pdf]

ADVANCED SCIENCE
Open Access

Supporting Information

for *Adv. Sci.*, DOI 10.1002/advs.202203239

Intermixing-Driven Surface and Bulk Ferromagnetism in the Quantum Anomalous Hall Candidate $MnBi_6Te_{10}$

Abdul-Vakhab Tcakaev, Bastian Rubrecht, Jorge I. Facio, Volodymyr B. Zabolotnyy, Laura T. Corredor, Laura C. Folkers, Ekaterina Kochetkova, Thiago R. F. Peixoto, Philipp Kagerer, Simon Heinze, Hendrik Bentmann, Robert J. Green, Pierluigi Gargiani, Manuel Valvidares, Eugen Weschke, Maurits W. Haverkort, Friedrich Reinert, Jeroen van den Brink, Bernd Büchner, Anja U. B. Wolter, Anna Isaeva and Vladimir Hinkov**

Supporting Information for "Intermixing-driven surface and bulk ferromagnetism in the quantum anomalous Hall candidate $MnBi_6Te_{10}$"

Abdul V. Tcakaev,[1,2] Bastian Rubrecht,[3] Jorge I. Facio,[3,4] Volodymyr B. Zabolotnyy,[1,2] Laura T. Corredor,[3] Laura C. Folkers,[5,2] Ekaterina Kochetkova,[3] Thiago R. F. Peixoto,[6,2] Philipp Kagerer,[6,2] Simon Heinze,[7] Hendrik Bentmann,[6,2] Robert J. Green,[8,9] Pierluigi Gargiani,[10] Manuel Valvidares,[10] Eugen Weschke,[11] Maurits W. Haverkort,[7] Friedrich Reinert,[6,2] Jeroen van den Brink,[3,12,2] Bernd Büchner,[3,5,2] Anja U. B. Wolter,[3,2] Anna Isaeva,[13,3,*] and Vladimir Hinkov[1,2,†]

[1] *Physikalisches Institut (EP-IV), Fakultät für Physik und Astronomie, Universität Würzburg, Am Hubland, D-97074 Würzburg, Germany*
[2] *Würzburg-Dresden Cluster of Excellence ct.qmat, Germany*
[3] *Leibniz Institut für Festkörper- und Werkstoffforschung (IFW) Dresden, Helmholtzstraße 20, D-01069 Dresden, Germany*
[4] *Centro Atómico Bariloche, Instituto de Nanociencia y Nanotecnología (CNEA-CONICET) and Instituto Balseiro. Av. Bustillo 9500, Bariloche (8400), Argentina.*
[5] *Institut für Festkörper- und Materialphysik, Technische Universität Dresden, 01062 Dresden, Germany*
[6] *Physikalisches Institut (EP-VII), Fakultät für Physik und Astronomie, Universität Würzburg, Am Hubland, D-97074 Würzburg, Germany*
[7] *Institute for Theoretical Physics, Heidelberg University, Philosophenweg 19, 69120 Heidelberg, Germany*
[8] *Department of Physics and Astronomy and Stewart Blusson Quantum Matter Institute, University of British Columbia, Vancouver, BC V6T 1Z4, Canada*
[9] *Department of Physics and Engineering Physics, University of Saskatchewan, SK S7N 5E2 Saskatoon, Canada*
[10] *ALBA Synchrotron Light Source, E-08290 Cerdanyola del Vallès, Barcelona, Spain*
[11] *Helmholtz-Zentrum Berlin für Materialien und Energie, Albert-Einstein-Straße 15, D-12489 Berlin, Germany*
[12] *Institut für Theoretische Physik, Technische Universität Dresden, D-01062 Dresden, Germany*
[13] *Van der Waals-Zeeman Institute, Department of Physics and Astronomy, University of Amsterdam, Science Park 904, 1098 XH Amsterdam, The Netherlands*

(Dated: February 1, 2023)

I. CRYSTAL GROWTH AND STRUCTURE REFINEMENT

An obstacle that needs to be overcome to synthesize $MnBi_6Te_{10}$ is the closeness of its melting point (588 °C) to the melting point of $MnBi_4Te_7$ (590 °C) [1]. This fact strongly hinders the selectivity of their crystal-growth by melt cooling and promotes kinetics-driven crystallization of competing phases. Consequently, it is possible that a synthesis aimed to yield $Mn_{0.85}Bi_{4.1}Te_7$ results in $Mn_{0.73}Bi_{6.18}Te_{10}$ crystals, as happened in this study. Even more curiously, a subsequent synthesis using the same batch composition and tempering route, produced $Mn_{0.85}Bi_{4.1}Te_7$ crystals. Similar problems with co-crystallization of $MnBi_6Te_{10}$ and $MnBi_8Te_{13}$ were reported in Ref. [2], where the experiment's outcome strongly depended on the subtle details of tempering. Our attempts to grow $MnBi_6Te_{10}$ crystals from the stoichiometric mixtures of the binaries tended to yield $MnBi_8Te_{13}$. Our EDX measurements of these crystals reproducibly show lower Mn contents (ca. 3.3–4.0 at. %) that correspond to sub-stoichiometric $MnBi_8Te_{13}$. Three such crystals were also characterized by PXRD and SQUID magnetometry (cf. FIG. 2(c) of the main text) and exhibited reproducible results.

Four $MnBi_6Te_{10}$ crystals (denoted as sample #1 – #4) were extracted from the same ingot (Fig. S1) and characterized by EDX as Mn-deficient with respect to the idealized chemical formula (Fig. S2).

After all measurements were completed, samples #1 – #4 were individually ground into powder and studied by X-ray diffraction. A full-range pattern of sample #2 is shown in FIG. S3. Out of all four samples, this one demonstrated the highest data intensity and is therefore discussed here. Other crystals delivered similar outcomes with typically lower fraction of Bi_2Te_3 admixture (2–3 wt. %). A Le Bail decomposition for the spectrum of sample #2 was conducted in JANA2006 by refining the lattice parameters and peak shape function against the measured data. Subsequently atoms were introduced for all phases [1, 2] and their positions refined via the Rietveld method. Isotropic displacement parameters were refined assuming the full occupancy of all atomic sites and then fixed to

* Corresponding address: a.isaeva@uva.nl
† Corresponding address: hinkov@physik.uni-wuerzburg.de

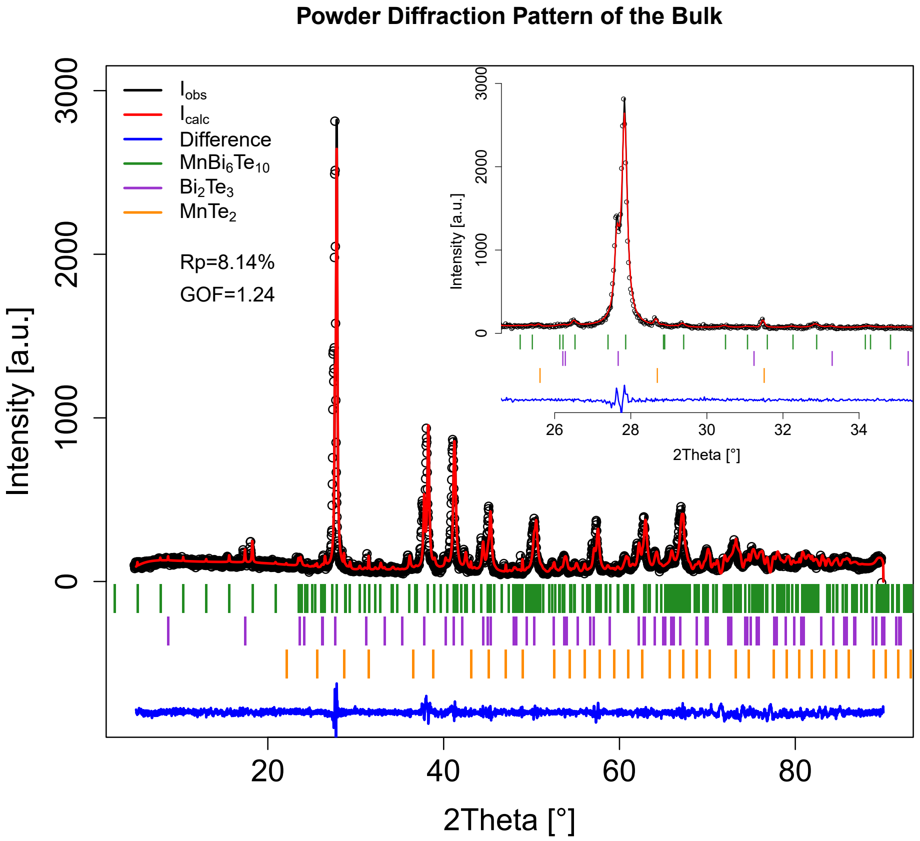

FIG. S 1. Experimental (black) and refined by the Le Bail method (red) PXRD patterns of the molten ingot, from which individual crystals were mechanically extracted. The difference curve is shown in blue (R_p = 0.081 %, R_{wp} = 0.109, GoF = 1.24); the vertical ticks mark the Bragg reflection positions for each identified phase. The sample is a three-phase mixture of $MnBi_6Te_{10}$ (sp. gr. $R\bar{3}m$, $a = 4.3667(2)$ Å, $c = 101.869(4)$ Å), Bi_2Te_3 (sp. gr. $R\bar{3}m$, $a = 4.3799(3)$ Å, $c = 30.491(2)$ Å) and $MnTe_2$ (sp. gr. $Pa\bar{3}$, $a = 6.9494(3)$ Å).

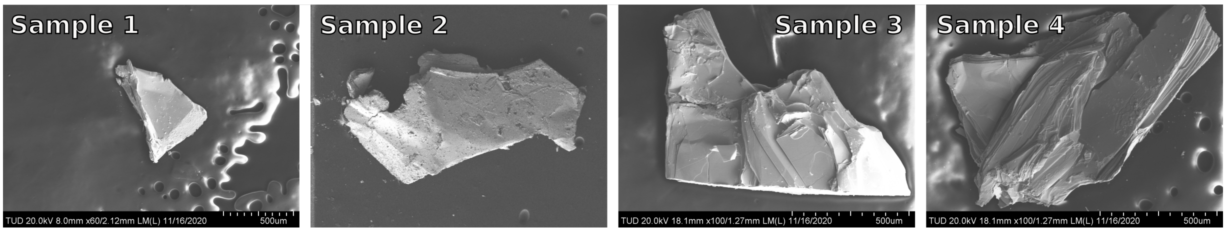

FIG. S 2. SEM images of the selected $MnBi_6Te_{10}$ crystals #1–4 that were used for the presented SQUID magnetization and XMCD experiments. The found chemical compositions (EDX) are as follows (in at. %): Sample #1: Mn 4.1(4), Bi 37.5(4), Te 58.40(4); Sample #2: Mn 4.6(2), Bi 37.8(8), Te 57.6(7); Sample #3: Mn 4.5(3), Bi 36.9(2), Te 58.6(1); Sample #4: Mn 4.4(6), Bi 36.6(7), Te 59.0(5).

$U_{iso} = 0.02$ Å^2 (all Te atoms) and to 0.01 Å^2 (all cations) before the next steps. A significant decrease in the R-values (from $R_{obs} = 0.17$ to $R_{obs} = 0.10$) was observed when the cation site occupancies in $MnBi_6Te_{10}$ were allowed to vary. An unrestrained refinement of the cation occupancies (yet without atomic intermixing) resulted in the unphysical $Mn_{1.30}Bi_{4.95}Te_{10}$ composition with an over-occupancy of Mn in the $3a$ site and an under-occupancy of Bi in all three $6c$ sites. At the next step, we allowed Mn/Bi intermixing in all cation positions. A free refinement of the mixed occupancies converged to an unrealistic chemical formula $Mn_{1.62}Bi_{4.96}Te_{10}$ with $R_{obs} = 0.09$. To ameliorate this, the subsequent refinements were conducted under the constraint of a fixed overall composition. Several compositions close to the independently obtained EDX results, *e.g.* $Mn_{0.76}Bi_{6.24}Te_{10}$ and $Mn_{0.8}Bi_{6.2}Te_{10}$, were tested and yielded similar intermixing patterns (see Tables SI, II, III). In general, all simulations considering mixed occupancies and Mn-substoichiometry yielded lower R-values than the atomically ordered structure. We see it as a convincing argument

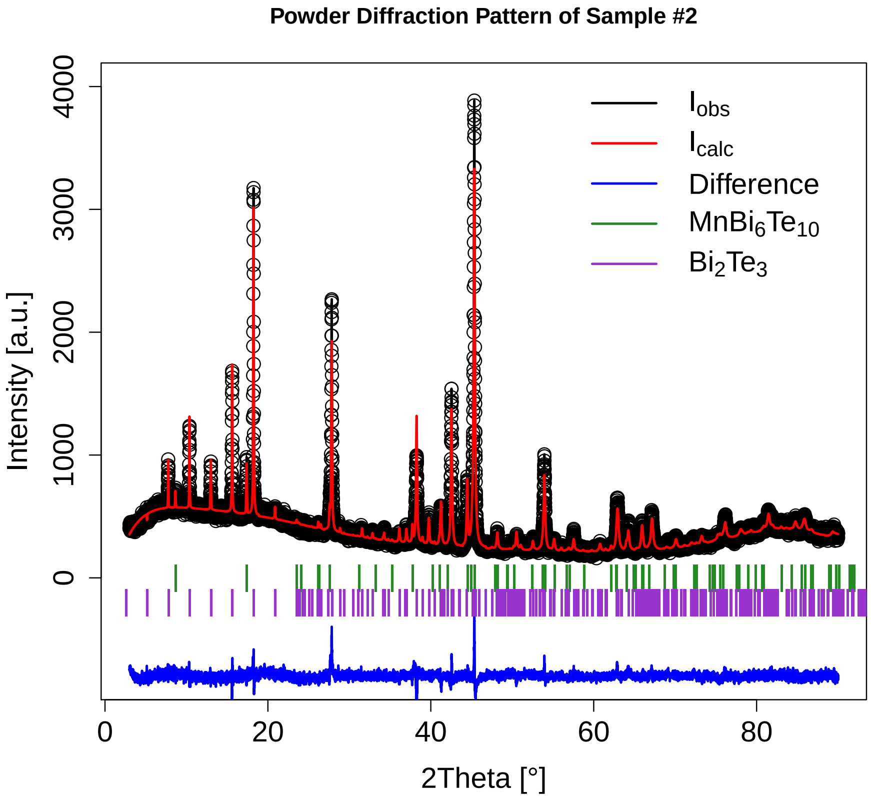

FIG. S 3. Experimental (black) and refined by the Rietveld method (red) PXRD patterns of the sample #2. The difference curve is shown in blue (R_p = 0.055, wR_p = 0.071, GoF = 1.48); the vertical ticks mark the Bragg reflection positions for each identified phase. A small fraction of 7 wt. % of Bi_2Te_3 is refined ($R\bar{3}m$, a = 4.3797(4) Å, c = 30.4965(7) Å, $R_{obs} = 0.094, wR_{obs} = 0.096, R_{all} = 0.109$). The refined parameters for the main phase $Mn_{0.8}Bi_{6.2}Te_{10}$ are given in Tables SI and III.

in favour of cation intermixing in the real structure of $MnBi_6Te_{10}$. However, due to the limited intensity of data obtained from the small specimen size as described above, we cannot narrow the refinement down to a statistically unequivocal quantification.

Nevertheless, we observed repeating outcomes in all considered refinement models with a cation disorder (cf. Tables SII, III): (1) According to our Refinements the SL contain between 44–48 % of Bi on the 3a site and up to 2% of Mn in the 6c site. (2) Furthermore, according to our refinements, the QLs contain 4–7 % of Mn on both Bi 6c positions.

II. MAGNETIZATION CHARACTERIZATION

In order to check the reproducibility of the magnetometry data on all four single crystals used in this study, we have performed field- and temperature-dependent measurements on samples # 1–4, see FIG. S4. All four crystals exhibit very similar magnetic properties, showing a ferromagnetic loop opening consistent with a quasi-saturated state with coercive fields at $T = 2$ K in the ∼ 32–42 mT range and a finite remanence at zero magnetic field, as discussed in the main text.

The bottom panel of FIG. S4 shows the field- and zero-field-cooled normalized magnetization M/H for a temperature range of 1.8 K to 30 K in an out-of-plane external magnetic field of 10 mT. A phase transition with a strong increase of the absolute value M/H is observed at around $T_c = (12.0 \pm 0.1)$ K, determined by the inflection point, as well as a notable FC/ZFC splitting around 10 K. These observations are in contrast with an antiferromagnetic transition at $T_N \sim 11$ K of $MnBi_6Te_{10}$ reported by other authors [3–6], which point towards possible differences in the Mn concentration and the Mn distribution between samples, such as the concentration of Mn/Bi intermixing due to the different growth conditions, see Section I.

Crystal system, space group	Trigonal, $R\bar{3}mH$ (No. 166)
Lattice parameters a, c (Å)	4.36778(8), 101.8326(6)
Cell volume (Å^3)	1682.44(2)
Formula units Z	3
M_r	2615.4
Density ρ (g cm^{-3})	7.75
Absorption μ (mm^{-1})	199.632
Temperature (K)	298
Radiation	Cu-K$_{\alpha 1}$ ($\lambda = 1.54056$ Å)
Range of collection	$3 \leq 2\theta \leq 71$, step = 0.007
No. of points	13254
Refined parameter	14
R_p, wR_p	0.055, 0.071
R_{obs}, wR_{obs}	0.079, 0.074
GooF	1.48

Table S I. Crystallographic data for the $Mn_{0.8}Bi_{6.2}Te_{10}$ model from the Rietveld refinement of Sample #2.

Atom	Site	x/a	y/b	c/z	U_{eq}	Occupancy
Mn1	$3a$	0	0	0	17.2	0.562(4)
Bi1	$3a$	0	0	0	17.2	0.442(4)
Mn2	$6c$	0	0	0.29684(1)	3.2	0.019(3)
Bi2	$6c$	0	0	0.29684(1)	3.2	0.981(3)
Mn3	$6c$	0	0	0.23650(1)	8.8	0.061(2)
Bi3	$6c$	0	0	0.23650(1)	8.8	0.939(2)
Mn4	$6c$	0	0	0.46984(1)	14.8	0.041(4)
Bi4	$6c$	0	0	0.46984(1)	14.8	0.959(4)
Te1	$6c$	0	0	0.34934(2)	17.7	1
Te2	$6c$	0	0	0.05416(3)	17.7	1
Te3	$6c$	0	0	0.41214(3)	17.7	1
Te4	$6c$	0	0	0.11676(3)	17.7	1
Te5	$6c$	0	0	0.17883(3)	17.7	1

Table S II. Atomic coordinates, site occupancies and isotropic displacement parameters (Å^2 $\times 10^3$) for the $Mn_{0.8}Bi_{6.2}Te_{10}$ model. U_{eq} is defined as 1/3 of the trace of the orthogonalised U_{ij} tensor. Mn1,2 and Bi1,2 belong to the SL, Mn3,4 and Bi3,4 belong to the QLs.

III. BULK DFT (GGA+U) CALCULATIONS

A. Intermixing modelling of $MnBi_2Te_4$

We have performed scalar relativistic calculations for various structural and magnetic models for $MnBi_2Te_4$. We have worked with a 2×2 supercell along the in-plane lattice vectors and the structural and magnetic models shown in Fig. S5. In addition to the defect-free case (S_0), we consider a single Mn vacancy (S_1), two Bi/Mn antisite defects (S_2 and S_3) and one antisite defect plus an additional Mn in the outer layer ($6c$ site of the SL block, S_4 and S_5). Models S_2 and S_3, as well as models S_4 and S_5, differ in the relative positions of the Bi atom in the central layer and the Mn atom in the Bi layer. For each structural model, we consider four possible magnetic arrangements. All models have in common that Mn moments order FM within any given atomic layer, but differ in the magnetic arrangement between the atomic layers within a SL, and between the atomic layers in the adjacent SLs (Fig. 5b): In the model A(D), Mn are coupled AFM(FM) within each SL and also between the SLs. In the model B(C) the intralayer coupling is AFM (FM), while the interlayer coupling is FM(AFM).

Fig. S5a shows the Wigner-Seitz cells of the structural models considered. In addition to the defect-free case (S_0), we consider the following possible defects: a single Mn vacancy (S_1), two Bi/Mn antisite defects (S_2 and S_3) and one antisite defect plus an additional Mn in the outer layer (S_4 and S_5). Models S_4 and S_5 (and similarly S_2 and S_3) differ in the relative positions between the Sb in the central layer and the Mn in the Sb Layer. For each structural model, we consider four possible magnetic models considering the possibilities of FM or AFM intrablock ordering and FM or AFM interblock ordering. All models assume a FM order of Mn within a given atomic layer. By intrablock order we refer to the order between Mn in the central layer (made of pure Mn in the absence of defects) and the outer layer (made of pure Bi in the absence of defects) of the SL. Fig. S5b sketches the magnetic models. In model

Atom	Site	x/a	y/b	c/z	U_{eq}	Occupancy
Mn1	$3a$	0	0	0	17.2	0.526(4)
Bi1	$3a$	0	0	0	17.2	0.478(4)
Mn2	$6c$	0	0	0.29689(1)	10.0	0.005(3)
Bi2	$6c$	0	0	0.29689(1)	10.0	0.995(3)
Mn3	$6c$	0	0	0.23651(1)	8.8	0.068(2)
Bi3	$6c$	0	0	0.23651(1)	8.8	0.932(2)
Mn4	$6c$	0	0	0.46987(1)	17.2	0.046(4)
Bi4	$6c$	0	0	0.46987(1)	17.2	0.954(4)
Te1	$6c$	0	0	0.34934(2)	17.7	1
Te2	$6c$	0	0	0.05419(3)	17.7	1
Te3	$6c$	0	0	0.41215(3)	17.7	1
Te4	$6c$	0	0	0.11675(3)	17.7	1
Te5	$6c$	0	0	0.17882(3)	17.7	1

Table S III. Atomic coordinates, site occupancies and isotropic displacement parameters (Å^2 $\times 10^3$) for the $Mn_{0.76}Bi_{6.24}Te_{10}$ model. U_{eq} is defined as 1/3 of the trace of the orthogonalised U_{ij} tensor. Mn1,2 and Bi1,2 belong to the SL, Mn3,4 and Bi3,4 belong to the QLs.

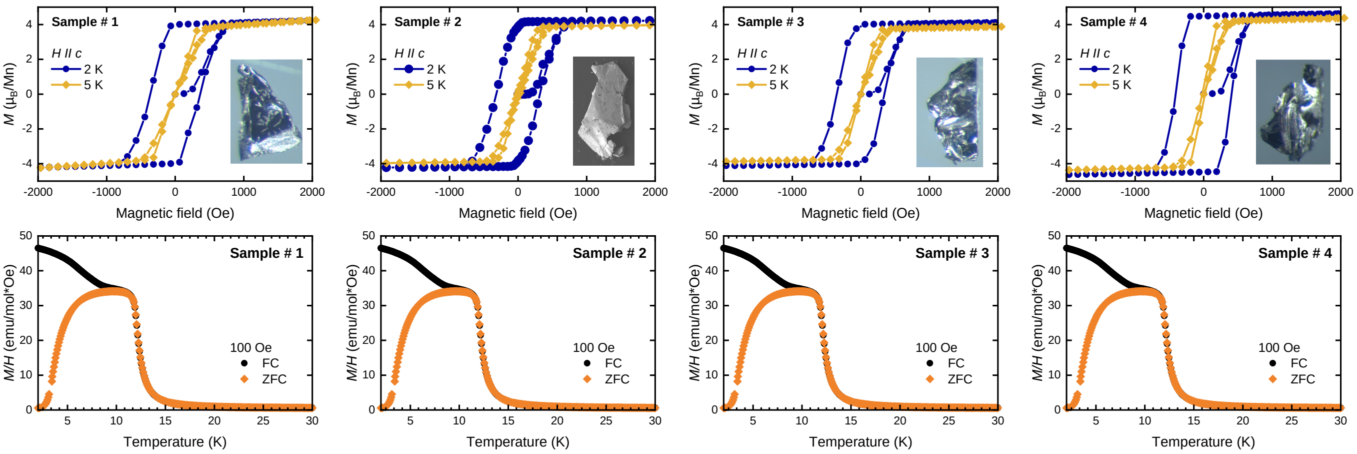

FIG. S 4. (Upper panel) Field-dependent magnetization curves taken on several crystals of $MnBi_6Te_{10}$ shown in the insets at 2 K and 5 K in an out-of-plane external magnetic field. No demagnetization correction has been applied. The absolute values of magnetization depend on the sample mass, which was about 10 times smaller for samples #1 and #4, thereby increasing the error, as well as on the real composition of each crystal. The Mn content in this series was evaluated by EDX yielding: 4.1(4) at. % Mn in Sample #1, 4.6(2) at. % Mn in Sample #2, 4.5(3) at. % Mn in Sample #3, and 4.4(6) at. % in Sample #4. (Bottom panel) Temperature-dependent normalized magnetization, measured with the zero-field cooled (ZFC) and field-cooled (FC) protocols (orange and black symbols respectively). The measurements were taken in an out-of-plane external magnetic field of 10 mT.

A (D), Mn are ordered AFM (FM) inside each SL and also between SLs. Model B (C) is AFM (FM) within a SL and FM (AFM) between SLs. Notice that we use experimental lattice parameters and that we will only compare total energies of different magnetic models for a given structural model. The main approximation associated with using experimental lattice parameters lies in assuming that possible strains due to the structural defects do not favor a particular magnetic order over the others.

The structural models considered have the inversion symmetry together with a reflection symmetry and one C_2 rotation and admit a monoclinic representation with the space group 12. We use this representation, shown in Fig. S5c for the model S_5. For models A and C we double the unit-cell along the Te-Te van der Waals direction. Scalar relativistic calculations were converged using a mesh of k-points of $16 \times 16 \times 8$ subdivisions ($16 \times 16 \times 4$ for the configurations which required doubling of the unit-cell).

Fig. S5d shows the energy difference Δ_{BA} between the structural models that assume the intralayer AFM order and the interlayer FM (model B) or AFM (model A). It can be seen that in general Δ_{BA} is reduced (the tendency towards antiferromagnetism is weakened), when structural defects are considered. Δ_{BA} varies significantly between the structural models, and in particular a FM order between the SLs is preferred for a configuration with additional Mn in the $6c$ site of the SL block. A similar conclusion can be reached for the models that assume the FM ordering

between the atomic layers of a SL (models C and D, Fig. S5e).

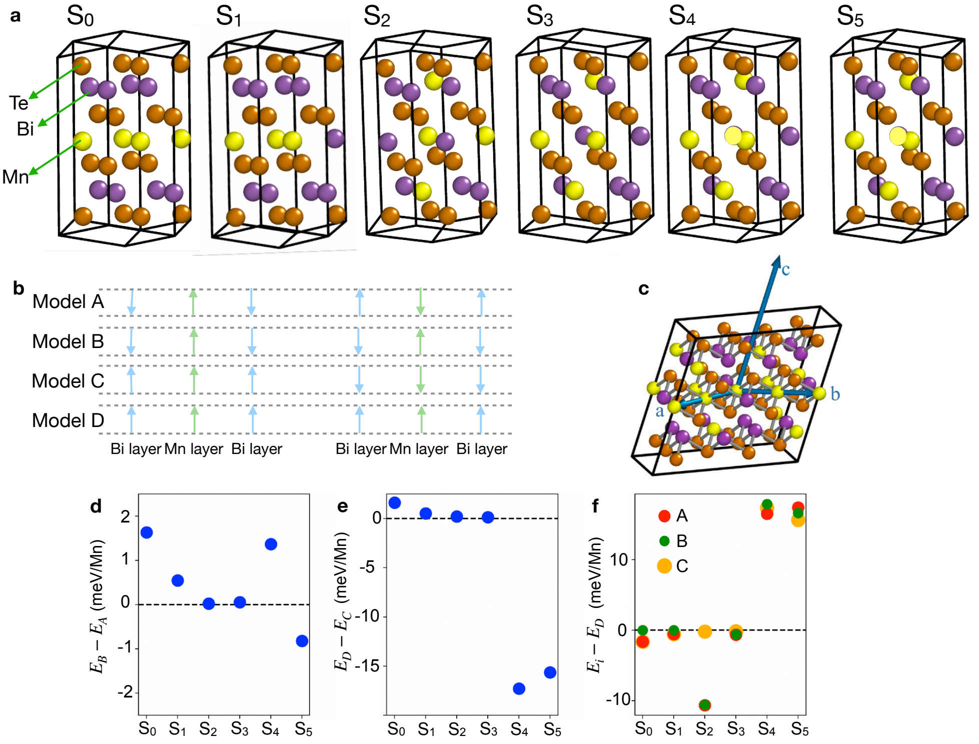

FIG. S 5. (a) The Wigner–Seitz cell of the considered structure models. (b) Magnetic models considered for each structure model. All magnetic models assume a ferromagnetic order with an out-of-plane orientation within each atomic layer. (c) The unit cell in the space group No. 12 for the S_5 case. (d) Difference between the total energies obtained from scalar-relativistic calculations for the magnetic models B and A. (e) Same as (d) for the magnetic models D and C. (f) Energy difference of all magnetic models with respect to model D for each structural model.

B. Structural models of intermixing for $MnBi_4Te_7$

Fig. S6 shows a side view of the 15 structural models considered for $MnBi_4Te_7$.

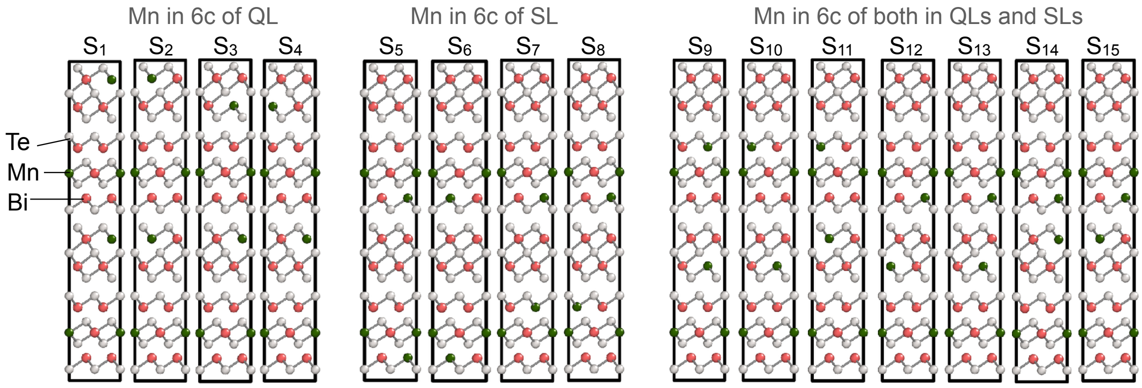

FIG. S 6. Side view (the *ac* plane) of the 15 structural defect models considered for $MnBi_4Te_7$. The view is the same as the one used in the schematic structure plots in Fig. 3 of the main text.

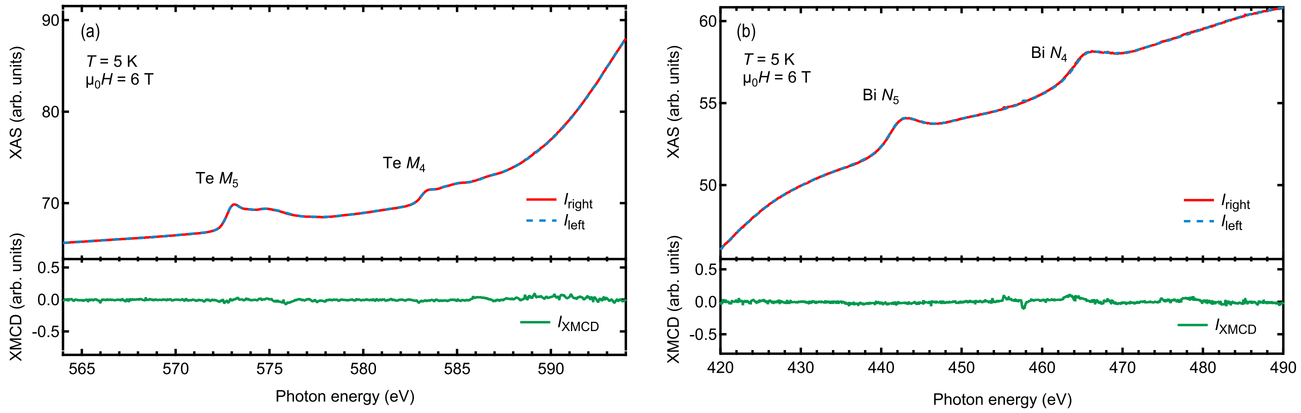

FIG. S 7. (a) Te $M_{4,5}$ edge XAS (TEY) spectra measured for left- ($I_{\rm left}$, blue curve) and right- ($I_{\rm right}$, red curve) circularly polarized x-rays (upper panel), together with the corresponding XMCD spectrum ($I_{\rm XMCD} = I_{\rm left} - I_{\rm right}$, bottom panel). (b) Bi $N_{4,5}$ edge XAS (TEY) spectra measured for left- (blue curve) and right- (red curve) circularly polarized x-rays (upper panel), together with the corresponding XMCD spectrum (bottom panel). All measurements were performed at $T \approx 5$ K and $\mu_0 H = 6$ T.

IV. TE AND BI XAS AND XMCD

Fig. S7 shows XAS and XMCD spectra measured around the Te $M_{4,5}$ and Bi $N_{4,5}$ absorption edges at $T \approx 5$ K in an applied magnetic field of 6 T. For V- and Cr-doped $(Bi,Sb)_2Te_3$, significant spin polarization was detected in XMCD at the nominally nonmagnetic Sb and Te host atoms [7–10]. However the data we present here for $MnBi_6Te_{10}$ exhibits no detectable spin polarization on the Te and Bi sites, which might be an indication of a different origin of the ferromagnetic interaction between Mn atoms within the septuple layer as compared to the ferromagnetic interaction between V and Cr impurities in $(Bi,Sb)_2Te_3$.

V. XMCD SUM RULE AND PEAK ASYMMETRY ANALYSIS

There are several ways to estimate the magnetic moment of Mn based on $L_{2,3}$ edge XAS and XMCD. Using the sum rules one can relate the intensities integrated over the L_3 and L_2 edges to the orbital and spin magnetic moments per Mn atom, $m_{\rm orb}$ and $m_{\rm spin}$, in the ground state. Alternatively, the Mn 3d spin magnetic moment can be obtained by determining the peak asymmetry of the experimental XMCD curve, $A = (I_{\rm left} - I_{\rm right})/(I_{\rm left} + I_{\rm right})$, i.e. the maximum of the L_3 XMCD divided by the maximum of the XAS summed over both polarizations, after suitable background correction of the XAS, and correlating this result with the calculated spectra of comparable line width. In the following section V A we provide details of the sum rule approach and its application to the x-ray data. Then, in section V B we describe the application of the peak asymmetry approach.

A. XMCD sum rule analysis for $MnBi_6Te_{10}$

In order to evaluate the orbital and the spin magnetic moments of Mn in our samples, as well as their ratio, we applied the sum rules to the $L_{2,3}$ edge XAS/XMCD spectra measured on sample #1 and sample #4. The sum rules relate in a quantitative manner the integrated XMCD and XAS of a specific shell to the ground-state orbital and spin magnetic moments,

$$m_{\rm orb} = -\frac{4}{3}(10 - n_d)\frac{\int_{L_{2,3}}(I_{\rm left} - I_{\rm right})\,dE}{\int_{L_{2,3}}(I_{\rm left} + I_{\rm right})\,dE} = -\frac{4}{3}\frac{q}{r}(10 - n_d), \tag{1}$$

$$m_{\rm spin} = -(10 - n_d)\frac{6\int_{L_3}(I_{\rm left} - I_{\rm right})\,dE - 4\int_{L_{2,3}}(I_{\rm left} - I_{\rm right})\,dE}{\int_{L_{2,3}}(I_{\rm left} + I_{\rm right})\,dE}C + 7\langle {\rm T}_z\rangle = -(10 - n_d)\frac{6p - 4q}{r}C + 7\langle {\rm T}_z\rangle, \tag{2}$$

$$\frac{m_{\text{orb}}}{m_{\text{spin}} - 7\langle \mathrm{T}_z \rangle} = \frac{1}{(9/2)(p/q) - 3}, \tag{3}$$

where p and q are the intensities of $(I_{\text{left}} - I_{\text{right}})$ integrated over the L_3 and over the $L_{2,3}$ edge, respectively, r is the intensity of $(I_{\text{left}} + I_{\text{right}})$ integrated over the $L_{2,3}$ edge, as shown in FIG. 6(c) of the main text, and n_d stands for the $3d$ level filling. $\langle \mathrm{T}_z \rangle$ is the expectation value of the intra-atomic magnetic dipole operator and C is a correction factor, which takes into account the jj mixing between $2p_{3/2}$ and $2p_{1/2}$ manifolds, arising from the relatively small spin–orbit coupling in the $2p$ shell of light transition metals. In the last equation the orbital to spin magnetic moment ratio is expressed as a function of only the branching ratio, p/q, of the XMCD signal. We use a value of $n_h = 4.7$, i.e., assuming a $3d$ electron filling of 5.3, inferred from the multiplet ligand-field theory calculations (MLFT), as well as from the DFT results.

Furthermore, the calculation of the spin magnetic moment requires the value of $\langle \mathrm{T}_z \rangle$. Many authors often neglect this term for $3d$ electrons, arguing that it results only in a small correction. The following estimation of $\langle \mathrm{T}_z \rangle$ done for Mn by means of MLFT shows that in the particular case of Mn with predominant d^5 configuration, its contribution to the m_{spin} is indeed negligible. We calculate the magnetic dipole operator according to the following equation:

$$\mathbf{T} = \sum_i \mathbf{S}_i - 3\mathbf{r}_i(\mathbf{r}_i \cdot \mathbf{S}_i), \tag{4}$$

where i runs over all electrons [11]. One only needs the value of T_z. We write it in spherical harmonics:

$$\mathrm{T}_z = \sum_i (1 - 3\hat{z}^2)\mathrm{S}_z^i - 3\hat{z}\hat{x}\mathrm{S}_x^i - 3\hat{z}\hat{y}\mathrm{S}_y^i = \sum_i -2C_0^{(2)}\mathrm{S}_z^i - \frac{1}{2}\sqrt{6}(-C_1^{(2)}\mathrm{S}_i^- + C_{-1}^{(2)}\mathrm{S}_i^+), \tag{5}$$

where $C_m^k(\theta,\phi) = \sqrt{\frac{4\pi}{2l+1}}Y_m^k(\theta,\phi)$ are renormalized spherical Harmonics, $\mathrm{S}_x = \frac{1}{2}(\mathrm{S}^+ + \mathrm{S}^-)$, $\mathrm{S}_y = \frac{i}{2}(-\mathrm{S}^+ + \mathrm{S}^-)$.

Knowing the ground state of the system from our MLFT calculations we can readily estimate the expectation value of the magnetic dipole term to be $\langle \mathrm{T}_z \rangle = -0.0002$. In Eqs. (1–5) all magnetic moments are measured in Bohr magnetons and the angular momenta in units of $\hbar$.

The applicability of the spin sum rules critically depends on how well the contributions of the L_3 ($j = 3/2$) and L_2 ($j = 1/2$) edges are separated in energy. Due to the strong jj mixing arising from the $2p-3d$ Coulomb interaction and the relatively small spin–orbit coupling in the $2p$ shell, there is a considerable overlap of these contributions for transition metal elements. Therefore, in our spectra the cutoff energy $E_{\text{cutoff}} = 648$ eV separating the $j = 3/2$ and $j = 1/2$ contributions is in principle ill-defined. The correction factor C is introduced to compensate the resulting deviation. To obtain C, here we followed the approach of Refs. [12, 13]. We formally applied the spin sum rule to our MLFT-based theoretical spectrum and derived $C = 1.40$ for Mn by comparison with the spin moment directly calculated for the ground state of the same model. The derived value of C is close to 1.47, which was estimated for (Ga,Mn)As having similar Mn $L_{2,3}$ XAS and XMCD line shapes [12].

Moreover, a significant source of error in our estimation of the magnetic moments, especially of the orbital one, is the subtraction of the background and edge steps, which is an issue due to the overlap of the Mn $L_{2,3}$ edges with the Te $M_{4,5}$ signal, and which is more problematic for $MnBi_6Te_{10}$ than for $MnBi_4Te_7$ and $MnBi_2Te_4$ due to the proportionally larger Te content. Fig. 6 (a) of the main text shows the Mn $L_{2,3}$ XAS signal $= I_{\text{left}} + I_{\text{right}}$ measured at $T \approx 3.5$ K in a field of 0.15 T, together with the background subtracted to obtain the resonant part of the spectrum.

Prior to the sum rule analysis we normalize the left- and right-circularly polarized spectra at energies far from the resonances. After that, we subtract the background indicated in Fig. 6 (a) (main text) from the XAS. The background-corrected XAS together with the XMCD is shown in FIG. 6 (b). The spectral part far from the resonance (between 620–634 eV and 660–670 eV) is manually set to zero. Further, we apply the sum rules 16384 times by randomly varying all parameters entering the analysis (such as n_d, C, $\langle \mathrm{T}_z \rangle$, integration energy ranges, normalization energy range) around their optimal values. Thus we get statistical distributions of m_{spin}, m_{orb} and $m_{\text{orb}}/m_{\text{spin}}$ for Mn as illustrated in Fig. 6 (d) (main text) for m_{spin}. This way, we are able to account for possible conjoined effects of the input parameters and produce fair estimates for the uncertainties in m_{spin}, m_{orb} and $m_{\text{orb}}/m_{\text{spin}}$. Furthermore, we include the estimate of the error bars coming also from various background correction approaches, including a simple linear background under L_3 and L_2, as well as a Shirley background.

B. XAS/XMCD peak asymmetry analysis to estimate the spin magnetic moment

Since XMCD intensity scales with the magnetization M, whereas the isotropic XAS intensity remains constant, an alternative method to obtain the spin magnetic moment of the Mn is to determine the asymmetry of the experimental

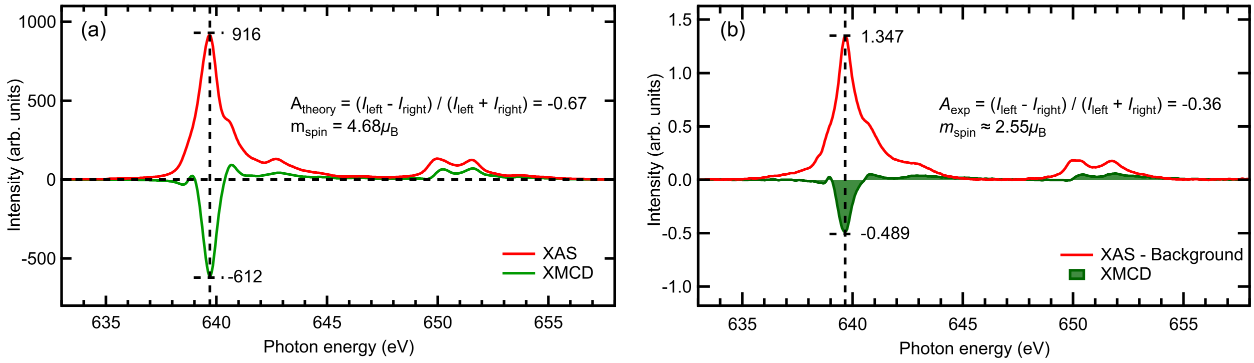

FIG. S 8. The asymmetry analysis of $MnBi_6Te_{10}$ (sample #4) XAS/XMCD spectra measured at $T \approx 3.5$ K in 0.15 T field. (a) Peak asymmetry $A_{\rm theory}$ for calculated XAS and XMCD spectra with 100% circularly polarized x-rays at the Mn L_3 absorption edge. The values of $(I_{\rm left} - I_{\rm right})$ and $(I_{\rm left} + I_{\rm right})$ are indicated by dashed horizontal lines. (d) Peak asymmetry $A_{\rm exp}$ for measured XAS and XMCD spectra.

XMCD $A_{\rm exp} = (I_{\rm left} - I_{\rm right})/(I_{\rm left} + I_{\rm right})$ after a suitable background correction of the XAS and to compare this result with the theoretical value $A_{\rm theory}$ obtained via MLFT calculated spectra of comparable line width. This circumvents complications and ambiguities in the sum rule analysis, especially when there is a large jj mixing, as in the case of Mn. However, if there is an unaccounted fraction of non-magnetic or antiferromagnetic Mn, the magnetic moment will be reduced accordingly.

Fig. S8 (a) shows that at the L_3 maximum the calculated Mn has an asymmetry $A_{\rm theory} = -0.67$, which can be related to a ground-state spin moment of $4.68\mu_{\rm B}$/Mn. For the experimental spectra measured on Sample #4 in a magnetic field of 0.15 T we obtain an asymmetry at the Mn L_3 peak maximum equal to $A_{\rm exp} = -0.36$. Comparing this value to the theoretical value, we obtain the ordered moment, i. e. the projection of the Mn magnetic moment on the magnetization direction (along the c axis), $m_{\rm spin} = \frac{-0.36}{-0.67} * 4.68\mu_{\rm B} \approx 2.55\mu_{\rm B}$/Mn in 0.15 T magnetic field, which is 54% of the calculated local Mn magnetic moment.

[1] D. Souchay, M. Nentwig, D. Günther, S. Keilholz, J. de Boor, A. Zeugner, A. Isaeva, M. Ruck, A. U. B. Wolter, B. Büchner, and O. Oeckler, J. Mater. Chem. C **7**, 9939 (2019).

[2] C. Hu, L. Ding, K. N. Gordon, B. Ghosh, H.-J. Tien, H. Li, A. G. Linn, S.-W. Lien, C.-Y. Huang, S. Mackey, J. Liu, P. V. S. Reddy, B. Singh, A. Agarwal, A. Bansil, M. Song, D. Li, S.-Y. Xu, H. Lin, H. Cao, T.-R. Chang, D. Dessau, and N. Ni, Sci. Adv. **6** (2020), 10.1126/sciadv.aba4275.

[3] I. I. Klimovskikh, M. M. Otrokov, D. Estyunin, S. V. Eremeev, S. O. Filnov, A. Koroleva, E. Shevchenko, V. Voroshnin, A. G. Rybkin, I. P. Rusinov, M. Blanco-Rey, M. Hoffmann, Z. S. Aliev, M. B. Babanly, I. R. Amiraslanov, N. A. Abdullayev, V. N. Zverev, A. Kimura, O. E. Tereshchenko, K. A. Kokh, L. Petaccia, G. Di Santo, A. Ernst, P. M. Echenique, N. T. Mamedov, A. M. Shikin, and E. V. Chulkov, npj Quantum Mater. **5**, 54 (2020).

[4] S. Tian, S. Gao, S. Nie, Y. Qian, C. Gong, Y. Fu, H. Li, W. Fan, P. Zhang, T. Kondo, S. Shin, J. Adell, H. Fedderwitz, H. Ding, Z. Wang, T. Qian, and H. Lei, Phys. Rev. B **102**, 035144 (2020).

[5] M. Z. Shi, B. Lei, C. S. Zhu, D. H. Ma, J. H. Cui, Z. L. Sun, J. J. Ying, and X. H. Chen, Phys. Rev. B **100**, 155144 (2019).

[6] J.-Q. Yan, Y. H. Liu, D. S. Parker, Y. Wu, A. A. Aczel, M. Matsuda, M. A. McGuire, and B. C. Sales, Phys. Rev. Materials **4**, 054202 (2020).

[7] A. Tcakaev, V. B. Zabolotnyy, R. J. Green, T. R. F. Peixoto, F. Stier, M. Dettbarn, S. Schreyeck, M. Winnerlein, R. C. Vidal, S. Schatz, H. B. Vasili, M. Valvidares, K. Brunner, C. Gould, H. Bentmann, F. Reinert, L. W. Molenkamp, and V. Hinkov, Phys. Rev. B **101**, 045127 (2020).

[8] M. F. Islam, C. M. Canali, A. Pertsova, A. Balatsky, S. K. Mahatha, C. Carbone, A. Barla, K. A. Kokh, O. E. Tereshchenko, E. Jiménez, N. B. Brookes, P. Gargiani, M. Valvidares, S. Schatz, T. R. F. Peixoto, H. Bentmann, F. Reinert, J. Jung, T. Bathon, K. Fauth, M. Bode, and P. Sessi, Phys. Rev. B **97**, 155429 (2018).

[9] M. Ye, T. Xu, G. Li, S. Qiao, Y. Takeda, Y. Saitoh, S.-Y. Zhu, M. Nurmamat, K. Sumida, Y. Ishida, S. Shin, and A. Kimura, Phys. Rev. B **99**, 144413 (2019).

[10] M. Ye, W. Li, S. Zhu, Y. Takeda, Y. Saitoh, J. Wang, H. Pan, M. Nurmamat, K. Sumida, F. Ji, Z. Liu, H. Yang, Z. Liu, D. Shen, A. Kimura, S. Qiao, and X. Xie, Nat. Commun. **6**, 8913 (2015).

[11] M. W. Haverkort, Spin and orbital degrees of freedom in transition metal oxides and oxide thin films studied by soft x-ray absorption spectroscopy, PhD thesis, Universität zu Köln (2005).

[12] K. W. Edmonds, N. R. S. Farley, T. K. Johal, G. van der Laan, R. P. Campion, B. L. Gallagher, and C. T. Foxon, Phys. Rev. B **71**, 064418 (2005).

[13] C. Piamonteze, P. Miedema, and F. M. F. de Groot, J. Phys. Conf. Ser. **190**, 012015 (2009).
